# Position statement on nutrition therapy for overweight and obesity: nutrition department of the Brazilian association for the study of obesity and metabolic syndrome (ABESO—2022)

**DOI:** 10.1186/s13098-023-01037-6

**Published:** 2023-06-09

**Authors:** Renata Bressan Pepe, Ana Maria Lottenberg, Clarissa Tamie Hiwatashi Fujiwara, Mônica Beyruti, Dennys Esper Cintra, Roberta Marcondes Machado, Alessandra Rodrigues, Natália Sanchez Oliveira Jensen, Ana Paula Silva Caldas, Ariana Ester Fernandes, Carina Rossoni, Fernanda Mattos, João Henrique Fabiano Motarelli, Josefina Bressan, Juliana Saldanha, Lis Mie Masuzawa Beda, Maria Sílvia Ferrari Lavrador, Mariana Del Bosco, Patrícia Cruz, Poliana Espíndola Correia, Priscila Maximino, Silvia Pereira, Sílvia Leite Faria, Silvia Maria Fraga Piovacari

**Affiliations:** 1 Grupo de Obesidade e Sindrome Metabolica, Hospital das Clinicas HCFMUSP, Faculdade de Medicina, Universidade de São Paulo, Sao Paulo, SP Brazil; 2 Laboratório de Lipides (LIM10), Hospital das Clínicas, Faculdade de Medicina, Universidade de São Paulo (HCFMUSP), São Paulo, SP Brazil; 3 Brazilian Association for the Study of Obesity and Metabolic Syndrome (ABESO), São Paulo, SP Brazil; 4 Centro de Estudos em Lipídios e Nutrigenômica – CELN – University of Campinas, Campinas, SP Brazil; 5 Liga Acadêmica de Controle de Diabetes do Hospital das Clínicas da Faculdade de Medicina da Universidade de São Paulo (HCFMUSP), São Paulo, SP Brazil; 6 Department of Nutrition and Health, Universidade Federal de Viçosa, Viçosa, MG Brazil; 7 Instituto de Saúde Ambiental, Faculdade de Medicina da Universidade de Lisboa, Lisbon, Portugal; 8 Programa de Obesidade e Cirurgia Bariátrica do Hospital Universitário Clementino Fraga Filho da UFRJ, Rio de Janeiro, RJ Brazil; 9 Núcleo de Estudos e Extensão em Comportamento Alimentar e Obesidade (NEPOCA) da Universidade de São Paulo - FMRP/USP, Ribeirão Preto, Brazil; 10 Universidade Federal Fluminense, Rio de Janeiro, RJ Brazil; 11 Universidade Federal do Rio Grande do Sul, Porto Alegre, RS Brazil; 12 Instituto PENSI - Fundação José Luiz Egydio Setúbal, Instituto Pensi, Fundação José Luiz Egydio Setúbal, Hospital Infantil Sabará, São Paulo, SP Brazil; 13 Núcleo de Saúde Alimentar da Sociedade Brasileira de Cirurgia Bariátrica e Metabólica, São Paulo, Brazil; 14 Gastrocirurgia de Brasilia/University of Brasilia, Brasilia, DF Brazil; 15 Hospital Israelita Albert Einstein, São Paulo, SP Brazil; 16 Nutrition Department of the Brazilian Association for the Study of Obesity and Metabolic Syndrome (ABESO), Rua Mato Grosso 306 – cj 1711, Sao Paulo, SP 01239-040 Brazil

**Keywords:** Obesity, Overweight, Weight loss, Nutrition assessment, Nutrition therapy, Diet, Dietary patterns, Nutritional counseling

## Abstract

Obesity is a chronic disease resulting from multifactorial causes mainly related to lifestyle (sedentary lifestyle, inadequate eating habits) and to other conditions such as genetic, hereditary, psychological, cultural, and ethnic factors. The weight loss process is slow and complex, and involves lifestyle changes with an emphasis on nutritional therapy, physical activity practice, psychological interventions, and pharmacological or surgical treatment. Because the management of obesity is a long-term process, it is essential that the nutritional treatment contributes to the maintenance of the individual’s global health. The main diet-related causes associated with excess weight are the high consumption of ultraprocessed foods, which are high in fats, sugars, and have high energy density; increased portion sizes; and low intake of fruits, vegetables, and grains. In addition, some situations negatively interfere with the weight loss process, such as fad diets that involve the belief in superfoods, the use of teas and phytotherapics, or even the avoidance of certain food groups, as has currently been the case for foods that are sources of carbohydrates. Individuals with obesity are often exposed to fad diets and, on a recurring basis, adhere to proposals with promises of quick solutions, which are not supported by the scientific literature. The adoption of a dietary pattern combining foods such as grains, lean meats, low-fat dairy, fruits, and vegetables, associated with an energy deficit, is the nutritional treatment recommended by the main international guidelines. Moreover, an emphasis on behavioral aspects including motivational interviewing and the encouragement for the individual to develop skills will contribute to achieve and maintain a healthy weight. Therefore, this Position Statement was prepared based on the analysis of the main randomized controlled studies and meta-analyses that tested different nutrition interventions for weight loss. Topics in the frontier of knowledge such as gut microbiota, inflammation, and nutritional genomics, as well as the processes involved in weight regain, were included in this document. This Position Statement was prepared by the Nutrition Department of the Brazilian Association for the Study of Obesity and Metabolic Syndrome (ABESO), with the collaboration of dietitians from research and clinical fields with an emphasis on strategies for weight loss.

## Introduction

This Position Statement was prepared by the Nutrition Department of the Brazilian Association for the Study of Obesity and Metabolic Syndrome (ABESO), with the collaboration of dietitians from research and clinical fields with an emphasis on strategies for weight loss. The preparation of this document was based on results from the main randomized controlled studies and meta-analyses that tested different nutrition interventions for the treatment of obesity. In addition, we included the analysis and interpretation of studies with interventions that are not scientifically recognized and have a low evidence level, but that are of great popularity. We also addressed the topics of intestinal microbiota, inflammation and nutritional genomics in the context of obesity, as well as the processes involved in weight regain. Aspects related to eating behavior and nutritional strategies recommended for the adoption of adequate eating habits were also included in this Position Statement.

Obesity is a chronic, progressive disease resulting from multifactorial causes mainly related to lifestyle (sedentary lifestyle, inadequate eating habits) and to other conditions such as genetic, hereditary, psychological, cultural, and ethnic factors [1, 2]. The treatment is complex, long-lasting, and involves lifestyle changes with an emphasis on nutritional therapy, physical activity practice, psychological interventions, and pharmacological or surgical treatment. Due to the chronic character of obesity, the diet recommended for it’s treatment must contribute to the individual’s global health. Even a modest reduction in body weight (5%) can already improve the metabolic profile and reduce cardiovascular risk [3].

**Fig. 1:**
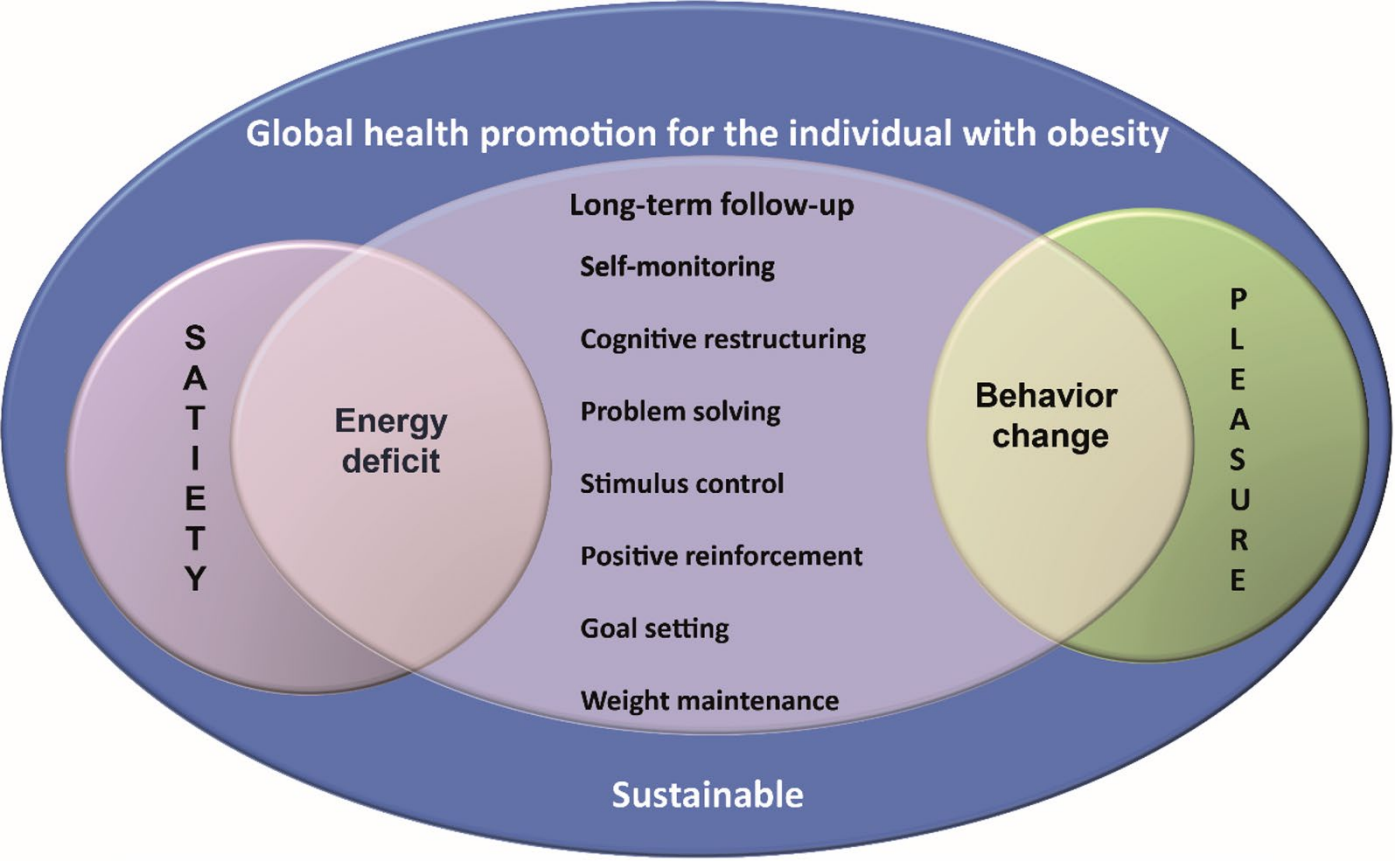
Strategies for successful long-term weight loss

Figure 1 shows examples of strategies for the long-term follow-up of patients with obesity, seeking global health.

The main diet-related cause associated with obesity observed in Brazil over the last decades was the increase in calorie intake, mostly associated with a gradual increase in the consumption of ultraprocessed foods. A cross-sectional study conducted in Brazil in 2008–2009, with 55.970 participants, showed that these products represent from 15.5% (lower quartile) to 39.4% (upper quartile) of the total dietary energy intake [4]. The greater household availability of these foods was positively and independently associated with a higher prevalence of excess weight and obesity in all age groups [4]. According to NHANES (National Health and Nutrition Examination Survey) data from the past 2 decades, the estimated proportion of energy intake from ultraprocessed foods has increased among youths in the United States and has contributed significantly to their total energy intake (TEI) [5]. Another important investigation conducted in several countries showed that ultraprocessed foods consumption ranged from 18% of TEI among preschool children in Colombia to 68% among adolescents in the United Kingdom. In almost all countries and age groups, the increase in ultraprocessed foods intake was a potential determinant of obesity in children and adolescents [6].

Low vegetable intake is known to be another relevant factor associated with obesity, as shown in a North American study conducted with 120,877 individuals followed during 4 years [7]. In this investigation, there was an inverse association between weight gain and the consumption of fruits, vegetables, whole-grains, and nuts [7]. In the Global Burden of Disease Study 2017 (GBD) [8], even though there was no specific analysis of the diet-related causes associated with obesity, this important study demonstrated the relationship between low intake of fiber and vegetables (fruits, grains and vegetables) and increased risk of cardiovascular morbidity and mortality.

In this scenario, the recommendation of a balanced diet for the prevention and treatment of obesity and its comorbidities is crucial. In the last years, the term “diet” has been associated with food restriction and highly restrictive meal plans that prioritize the exclusion of specific nutrients or food groups. The probable link between the word “diet” and food restriction might be associated with the use of the term “diet foods” in low-fat foods and sugar-free beverages [9], or even with the beginning of the use of artificial sweeteners in the mid-twentieth century [10]. Therefore, there was a negative reaction from the general population toward the adoption of an adequate diet, even a non-restrictive diet. Even some health professionals have opted not to prescribe diets for their patients. In this context, it is worth clarifying the real meaning of the word “diet”, which comes from Greek and has a broad meaning [11]. The Greek term projects its meaning as a synonym for quality of life, comprehending healthy eating from a qualitative and quantitative point of view, among other practices. According to the European Society for Clinical Nutrition and Metabolism (ESPEN), diet is one of the main determinants of an individual’s future health, especially for the prevention of cardiometabolic diseases and cancer [12]. Thus, ABESO understands that the recovery of the real meaning of the word “diet” can contribute to the adherence to a healthy diet by the population and relieve something that seems difficult to follow.

Fad diets cause a significantly negative impact on the treatment of obesity. These approaches are spread by bloggers and digital influencers, as well as by some health professionals. Among the approaches that promise rapid weight loss, there are several fad diets, methods and programs, such as probiotics, teas, thermogenic capsules, phytotherapy, supplements without scientific evidence, gluten-free or lactose-free products, coconut fat, among others. These products are ineffective and have potentially harmful effects on health, and are many times created with the only purpose of promoting the marketing of products with a clear conflict of interest biases. In this regard, ABESO has been giving frequent warnings to the population about approaches that promise a rapid solution for such a complex condition as obesity.

Therefore, the elaboration of an individualized healthy meal plan, respecting individual cultural habits, lifestyle, preferences and possibilities, is essential for weight loss and maintenance, facilitating easier adherence to treatment. In this sense, highly restrictive diets are difficult to follow and are associated with increased short and long-term dropouts, potentially leading to weight regain [13, 14].

**Fig. 2:**
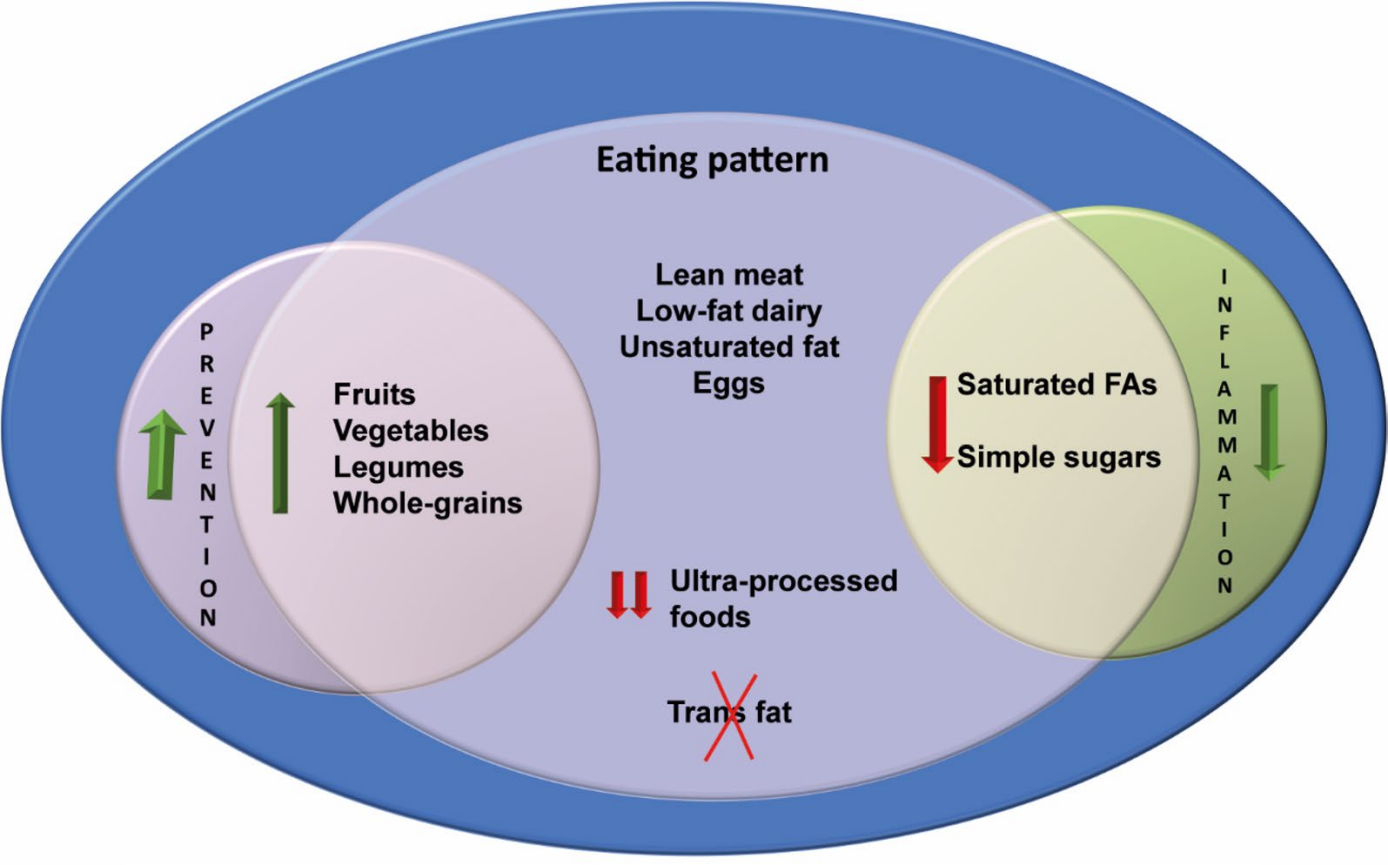
Healthy eating pattern for patients with obesity

The proposed treatment involves lifestyle changes, regular practice of physical activity and the adoption of a healthy dietary pattern that promotes an energy deficit to induce weight loss [2, 3]. Studies that compared different diets for weight loss showed that varied proportions of carbohydrates and fats had similar results in 12 months [13], demonstrating that the impact of calorie restriction is superior to that of macronutrient composition [15–18]. Nevertheless, the adoption of dietary patterns such as the Mediterranean diet [19–21] and the Dietary Approaches to Stop Hypertension (DASH) diet [22–24], mainly constituted by fruits, vegetables, grains, eggs, low-fat dairy and lean meats, can bring additional benefits by reducing cardiometabolic risk. Figure 2 shows factors of a healthy eating pattern for patients with obesity.

An important point to consider is the fact that nutritional guidance in isolation might not be sufficient to promote eating behavior change, since obesity is a cognitive behavioral condition [25]. Thus, the association of behavior therapy is essential for the development of abilities that will support the attainment of a healthy weight [26].

In this scenario, this Position Statement aims to discuss the main scientific evidence that may contribute to the clinical practice of health professionals regarding the nutritional treatment of obesity and reinforce the relevance of the adoption of an adequate diet that promotes energy deficit, that is sustainable in the long-term and that contributes to the individual’s global health. This document also emphasizes the importance of guiding dietitians on how to manage the nutritional treatment based on scientific evidence in addition to promoting eating behavior changes.

Box 1 presents a summary of nutrition recommendations for the treatment of overweight and obesity.

**Box 1:** Summary of nutrition recommendations for the treatment of overweight and obesity

Diet/Strategy	Statement	Class of recommendation	Level of evidence	Does ABESO recommend?
Energy density	Consumption of foods and beverages with low energy density associated with energy deficit from diet can be useful in the treatment of obesity	IIb	B	Y
Portion control	Portion size control can help to reduce the excessive consumption of foods and beverages	IIb	B	Y
Foods				
Fruits and vegetables	Encouraging the intake of fresh fruits and vegetables, associated with a hypocaloric diet, contributes to weight management	IIa	A	Y
Fast-food	The intake of fast-food meals high in sugars, sodium, and saturated and/or trans fat is not recommended	IIb	C	N
Ultraprocessed food	The consumption of ultraprocessed foods is associated with excessive calorie intake and weight gain	III	B	N
Sweetened beverages	Sweetened beverages or even unsweetened fruit juices contribute to increased calorie intake and body weight, and should be discouraged for the prevention and treatment of obesity	I	A	N
Calorie-based interventions				
Low-calorie diets	Low-calorie diets are fundamental for obesity treatment and the management of the meal plan must be associated with lifestyle changes	I	A	Y
Very low-calorie diets	VLCDs should not be the first option for obesity treatment	IIa	A	Specific situations
Meal replacements	Meal Replacements can help to structure a LCD and increase diet adherence	IIa	A	Y*
Based on dietary patterns				
Mediterranean	The Mediterranean diet has cardiovascular benefits to the individual with obesity	I	A	Y
Plant-based and vegetarian	Vegetarian or plant-based diets with reduced intake of ultraprocessed foods can be an option for the prevention and treatment of obesity	IIa	A	Y*
Based on macronutrients				
Low-carb	Low-carb diets promote weight loss in short and medium duration (3 to 6 months)	IIa	A	N
	Low-carb diets do not promote more weight loss compared to other diet types in long-term studies	IIa	A	
Ketogenic	The ketogenic diet should not be recommended for the treatment of obesity as it does not promote a balanced diet or favor adherence to healthy eating habits	III	A	N
Low-GI	Glycemic Index as a measure of carbohydrate quality appears to be a minor determinant for body weight, weight loss and obesity prevention	IIb	B	N
Based on				
Meal frequency and timing	Intermittent calorie restriction presents similar weight loss results as meal plans with continuous calorie restriction	IIb	A	N
	Weight loss is induced by the energy deficit, and not by the fasting period per se or the number of daily meals	IIb	A	
	Late consumption of most of the daily calories can negatively impact body weight	IIb	B	
Breakfast consumption	There is no conclusive evidence if breakfast plays a causal role both for weight gain and weight loss	IIb	A	Y
Sweeteners	There is inconclusive evidence about the effect on weight loss of sweeteners when used alone, without healthy food choices accompanied by an energy deficit	IIb	B	N*
Supplements				
Phytotherapics	There is no recommendation to use phytotherapics for weight loss	III	B	N
Caffeine	The use of caffeine supplements should not be considered as a strategy for weight loss and obesity treatment	III	B	N
Whey protein supplements	The prescription of whey protein is not indicated as a therapy for weight loss	III	A	N
Coconut fat	There is no scientific evidence to support the indication of coconut oil as a weight loss strategy	III	B	N
Probiotics	There is not enough scientific evidence for the use of probiotic supplementation for weight management	III	A	N
	There is not enough scientific evidence for performing gut microbiota tests in clinical practice for the treatment of obesity	III	A	N
Eating behavior				
Cognitive behavioral therapy	Cognitive-Bevahioral Therapy should be used for weight management in patients with overweight and obesity	I	A	Y
Motivational interviewing	Motivational Interviewing can be considered and used for weight management in patients with overweight and obesity	IIa	A	Y
Mindful eating	The results related to Mindfulness-Based Interventions in the treatment of obesity are still divergent, and it is yet not possible to guarantee that this is an appropriate approach for all patients	IIa	A	Y*

*Y* Yes, *N* No

^*^The strategy might be used as part of the hypocaloric diet; the individual’s profile must be always assessed

## Methods

For the preparation of the present position statement, the authors searched published scientific literature related to obesity with emphasis in nutrition therapy in PubMed, Scielo and Lilacs from January 1946 (comprising the concept and history of topics such as inflammation) to August 2022 and chose, whenever possible, evidence from randomized controlled trials, systematic reviews and meta-analyses published in high-impact journals, reading the full article to obtain information and performing a critical analysis.

The tables presenting the classes of recommendations and the levels of evidence in the present position statement using the following standards:

**Table:** 

Classes of recommendations	
Class I	There is evidence and general agreement favoring the recommendation
Class IIa	There is divergence, but the majority approves
Class IIb	There are divergence and conflicting opinions
Class III	Not recommended

Levels of evidence	
Level A	Multiple randomized controlled clinical trials
Level B	A single randomized controlled trial, non-randomized clinical trials or well-designed observational studies
Level C	The consensus opinion of experts

## Inflammation in obesity

In obesity, inflammatory mediators are systemically dispersed in the body, in levels slightly above the physiological threshold (low-grade inflammation), with the possibility of chronic permanence in the body [27, 28]. Although the mediators characteristic of obesity reaches a sufficient threshold to induce inflammation, their concentrations are not accurately detectable in routine biochemical tests [29].

Excessive intake of saturated fatty acids from the diet also initiate the inflammatory process when recognized by Toll-like receptors (TLRs) activating its downstream intracellular pathaway [30, 31]. Also, the high and constant consumption of refined carbohydrates is correlated with inflammatory stages and chronic diseases [32] due to de novo lipogenesis [33], which is induced by the excessive content of monosaccharides converted to saturated fatty acids [34], later directed to storage tissues [33]. An important note should be made regarding the consumption of high-protein diets, which also promote de novo lipogenesis [35]. Free fatty acids released from adipose tissue also contributes for TLR activation, such as, during intense lipolysis. The inflammation occurs when the visceral adipose tissue also becomes insulin resistant and induces lipolysis of triglycerides, with a high and constant release of free fatty acids into the bloodstream, turning inflammation into a systemic, redundant and perpetual condition [36–38].

During the weight gain process, the hypertrophic adipocytes increase the production of Tumor necrosis factor alpha (TNF-α), which assumes the character of a pro-inflammatory molecule and induces systemic insulin resistance [28]. Subsequently, studies have demonstrated proteins secreted by the adipose tissue, with influence on inflammation [39], especially monocyte chemoattraction proteins (MCP-1) [40] and interleukin 6 (IL-6) [41].

Thus, saturated fatty acids, when present in excessive amounts in the bloodstream, activate the downstream TLR pathway by recruiting the protein MyD88 (amyloid differentiation factor 88), which adapts extracellular to intracellular response [42]. From this, complex conformational changes occur in multiple subsequent proteins TRAF6 [TNF receptor-associated factor 6], TAB1/2 [TGFβ-activated kinase-binding protein] and TAK1 [TGFβ-activated kinase]) and culminate in the phosphorylation and activation of the IKK protein (kappa kinase inhibitor) [43, 44] and NF-kB (nuclear factor kappa B) protein [43, 44]. NF-kB migrates to the nucleus and initiates the transcription of genes controllers of pro-inflammatory proteins including TNF-α, IL1β, IL-6, IL-10, MCP-1 [27, 38, 45], among many others [46]. After TNF-α and IL1β receptors activation by its agonists, the inflammatory tonus is increased once their signals culminate in a redundant TAK1 activation [43, 44]. 

The permanence of the inflammatory state in the hypothalamic neuronal environment induces activation of the cell death program, with irreversible damage to the homeostasis of this important energy system [45].

The inflammatory process associated with obesity has a complex mechanism, and despite the great advances in understanding this phenomenon, there is still much to comprehend about its origin. Finding strategies capable of interfering with and blocking specific points of inflammatory signaling is essential for controlling the development of obesity, as well as the risk of related comorbidities.

From a nutritional standpoint, science is currently focused on what are the nutrients or other substances present in foods, what are their mechanisms of action, and, mainly, what is the amount consumed capable of implying a health risk, in terms of the induction of the inflammatory process characteristic of obesity. At the same time, evidence points to foods with less inflammatory or even anti-inflammatory potential, which could be more present in the population’s food habits, always considering and respecting the culture and food sovereignty of these populations.

**Table:** 

Statement	Level of evidence
1. Several compelling evidence demonstrates that inflammation is present in overweight and obese conditions	A
2. Several compelling evidence describes, in detail, the mechanisms of action of some saturated fatty acids, acting as initial inducers of the inflammatory process, in experimental and human models, when consumed in excessive amounts	A
3. There is evidence from experimental and human models that demonstrate that inflammation can occur even with the eutrophic state	C

## Nutritional genomics and obesity

Nutritional genomics comprises three main areas: nutrigenomics [47], nutrigenetics, and nutritional epigenetics [47–49]. It is hard to define which set of genes exert the most prominent influence on obesity or on the obesogenic process per se because, for each stage of the disease, a group of genes stands out. As an example, in the nutrigenomics field, studies using the resveratrol as a bioactive compound have described its ability to control intracellular signaling pathways involved with food intake and energy expenditure, among others [50]. The resveratrol group increased the activity of mitochondrial complex IV and the number of mitochondria in striated muscle. Although the study did not show beneficial effects on weight loss or insulin resistance, there was a clear demonstration of the action of the compound in the modulation of a very specific metabolic process [50].

Monogenic obesity is a rare condition. The most consolidated findings are the mutations in the leptin gene [51], in its receptor [52], and in proopiomelanocortin (POMC) neurotransmitters [53]. However, diseases can be also associated with mutations in several genes, being called polygenic diseases, such as the main chronic non-communicable diseases. In the field of nutrigenetics, the most studied polymorphisms are those that affect only a single nucleotide, called Single Nucleotide Polymorphism (SNP). Genome-wide association studies (GWAS) demonstrated an increasing array of possibilities about genetic interactions, their modifications, and interactions with environmental factors, in hundreds of thousands of individuals [54].

One of the most studied genes related to the search for polymorphisms associated with weight gain is the FTO (fat mass obesity) gene. Frayling et al. [55] identified in 13 cohorts, a variant in the FTO gene positively associated with high BMI in 16% of homozygous adults carrying the T > A risk allele (rs9939609). In Brazil, this same allele was associated with high body mass in children up to 8 years of age [56].

Taking the example of the FTO gene, even though the aforementioned variant (rs9939609) is the most commonly associated with the risk of weight gain, thousands of other variants of the FTO gene were identified, many still not having their clinical significance or relationship with diseases [57] well described and, at least 22, presenting an outcome showing benign clinical significance (https://www.ncbi.nlm.nih.gov/snp—filter: “*benign*”). Therefore, caution is required in the interpretation of the identified variant. Despite the many evidences about the rs9939609 variant, in the DPS study of T2DM prevention [58], a Finnish cohort with 522 middle-aged patients with overweight, and concluded that the presence of the risk allele did not modify weight change when lifestyle changes were adopted (diet and exercise). A meta-analysis of 218.166 adults and 19.268 children [59], showed that the association of the FTO risk allele with the odds of obesity was attenuated by 27% in physically active individuals. Likewise, in an arm of the PREDIMED (*Prevención con Dieta Mediterránea*) study [60] it was noticed that when allele-carriers and non-carriers were submitted to a 3-year intervention with a Mediterranean diet, the allele carriers had lower body weight gain than wild type subjects. This finding is attributed to the fact that the components of the Mediterranean diet (such as oleic acid, hydroxythyrosol, etc.) could compensate the negative effects of FTO on energy expenditure.

Other modifications were associated with weight gain, involving not only FTO, but also the HTR2C (serotonin receptor), PPAR-γ (peroxisome proliferator-activated receptor-gamma), LEP (leptin), LEPR (leptin receptor), ADRB2 (b2-adrenoreceptor), UCP1/2 (mitochondrial uncoupling protein-1 and 2), and FABP2 (fatty acid binding protein-2) genes, among others. From the range of identified variants, “genetic panels” have emerged, in an attempt to associate several polymorphic genes with the outcomes described in the literature. However, it must be clear that carrying a certain polymorphism does not mean that the phenotypic manifestation is mandatory, that is, it is not possible for an analysis to be deterministic. A recent study [17], an arm of the DIETFITS study (Diet Intervention Examining the Factors Interacting with Treatment Success), pre-selected 609 individuals carrying the polymorphism in the PPAR-γ (rs1801282), ADRB2 (rs1042714) or FABP2 (rs1799883) genes, in order to evaluate the interaction of these gene variants with two types of weight-loss diets. Subjects were randomized into two groups, receiving healthy diets, but low in fat or carbohydrates. After 12 months of intervention, none of the genotypes investigated showed any type of significant influence associated with weight change. Still, there was no interaction between the individuals' genotype and the type of diet offered [17]. Also, despite being a carrier of the risk allele, the disorder will hardly occur without the individual being exposed to the environmental characteristics that promote that disease [61].

Epigenetics studies how the chromatin condensation system, and, consequently, the gene, can be influenced by environmental factors (drugs, pollutants, pesticides, behavior, food, etc.). Briefly, histone acetylation leads to chromosome unfolding, while deacetylation suppresses it; and around the gene structure, while gene methylation suppresses its expression, demethylation favors it. Both events can occur independently and also be passed on to other generations. A given gene can be more or less expressed, modifying the individual's phenotype, even without any mutation associated with the gene.

Bera et al. [62], found that the epigenetic silencing of the ankyrin-26 (ANKRD26) gene induced weight gain in mice, while Desiderio et al. [63] correlated the high degree of its methylation with the increased body weight and expression of pro-inflammatory genes in the patients. Although the authors did not identify whether the methylation of the ANKRD26 gene was caused by obesity or an inherited trait, a study in rodents showed that the consumption of a diet rich in saturated fat induces methylation of the ANKRD26 gene [64].

The inheritance characteristics can have more control pathways than believed. Thrush et al. [65], after submitting 20 patients to weight loss with a restrictive diet with 900 kcal/day for 12 weeks, divided them into diet-sensitive or diet-resistant (those who did not lose weight). Later, all participants consumed a high-fat meal and had plasma samples collected every hour for 6 h. Exosomes were isolated and added to a culture media containing muscle cells (C2C12). Plasma exosomes from diet-resistant subjects showed decreased mitochondrial respiration and decreased fatty acid oxidation in the cells. It was demonstrated that diet-resistant individuals had lower amounts of heat shock protein 72 (HSP72), which is recognized for its role in energy metabolism, protecting from obesity [66].

Results of studies with follow-up of offspring at birth or in the first three years of life show little or no significance [67, 68] compared to studies that correlate the impact on maternal/paternal lifestyle with offspring over 20 years of age, so it seems that the main epigenetic manifestations occur from adolescence onwards [69]. In the “Lifeways Cross-Generation” cohort study [70], showed that not smoking, not drinking alcohol and maintaining a healthy diet during pregnancy correlated with the maintenance of normal weight in offspring when evaluated at 5 and 9 years of age, while the children of non-adherent mothers presented an inverse correlation [70].

Lastly, there is an urgent need for health professionals to improve the comprehension of the information obtained from the human genome, since many professionals are still not trained concerning the correct interpretations and instrumental methods, as well as companies that offer services of decoding and gene polymorphism analyses. Market speculation, associated with the anxiety of health professionals in the use of these tools can bring more difficulties to the patient's routine, with a harmful potential.

Despite the growing number of investigations about: (1) modifications in the pattern of gene expression induced by foods; (2) alterations in the pattern of response to nutrient metabolism according to genetic modifications between individuals; and (3) nutrient-induced pre- and postpartum changes in the structure and functioning of chromatin, defining the metabolic conditions of the offspring, this is a relatively new field and the knowledge gained from nutritional genomics is not yet a feasible approach to the treatment of obesity.

**Table:** 

Statement	Class of recommendation	Level of evidence
There is not sufficient scientific evidence for the use of “genetic test panels” for the treatment or prevention of obesity; therefore, its use is not recommended	III	A

## Gut microbiota and obesity

Diet plays a fundamental role on the composition, diversity and functionality of gut microbiota, which is associated with the obese/lean phenotypes in humans [71–75]. The low- grade inflammation observed in obesity relates to the LPS concentration, which plays a key role in the onset of obesity-related diseases [76].

Adherence to hypercaloric diets, high in fat and sugars, contributed to the loss of gut microbiota diversity, with important metabolic alterations [77–79]. Individuals with obesity present lower bacterial diversity, with a higher relative abundance of the Firmicutes genus and a decrease in Bacteroidetes [79–81], an effect potentiated by a high-fat diet [82]. The increase in Firmicutes is positively correlated with body fat and waist circumference and inversely correlated with lean mass [79]. Also, the decrease in bacterial diversity is associated with a more pronounced inflammatory profile, besides insulin resistance [75, 83, 84].

In obesity and/or in the presence of a high-fat diet, especially rich in saturated fatty acids, there is a decrease in the mucus layer [85], that is the first line of defense against invading enteric bacteria and LPS translocation. This probably occurs as a result of the reduction of *Akkermansia muciniphila*, a bacteria involved in the turnover of the mucus layer [86, 87] in the intestinal mucosa. In addition, a high-fat diet destabilizes tight junctions [85, 88], increasing intestinal permeability and favoring endotoxemia [89–93], associated with an increase in inflammatory biomarkers such as TNF, IL-1β and IL-6.

Saturated fatty acids, both from the diet and those present in the antigenic lipid A portion of LPS, are TLR ligands and induce inflammation in the intestinal cell, via NF-kB and NLR, increasing the synthesis of pro-inflammatory cytokines such as TNF-α, IL-1^®^ and IL-6 [94].

It is well established in the literature that the Western diet, characterized by a high consumption of fats and sugars and a low fiber intake, promotes unfavorable alterations in the composition of gut microbiota, resulting in dysbiosis, characterized by reduced bacterial diversity [95, 96], inducing endotoxemia [85, 91, 95–100]. Furthermore, this dietary pattern may lead to decrease in mucus layer thickness, which favored increased translocation of endotoxins, such as LPS [101–103].

In contrast, the adherence to balanced dietary patterns with fiber-rich foods favor a healthy gut microbiota profile. Bacteria obtain energy from fiber through the fermentation of polysaccharides, forming short-chain fatty acids (SCFAs), mainly acetate, butyrate, and propionate [95]. SCFAs decrease inflammation, improve insulin sensitivity, increase satiety, resulting in better glycemic control and weight control [104].

Although dietary fibers favor the development of a healthy microbiota, this benefit may be lost in the presence of a high-fat diet [105]. In addition to diet composition, caloric changes can also modify the composition of the gut microbiota. A short-term study in lean individuals and individuals with obesity showed that the increase in caloric load was able to interfere with the relative abundance of the two dominant bacterial phyla, Firmicutes and Bacteroidetes [106].

The benefit of moderate calorie restriction improved dysbiosis in individuals with a BMI > 25 kg/m^2^ on a Mediterranean diet [107] for a period of 3 months. Regardless of the weight loss observed by reducing calories, adherence to the Mediterranean diet promotes a healthy microbiota profile and contributes to the integrity of the intestinal barrier [108].

Growing evidence shows that maintaining an adequate weight, with calorie restriction when necessary [107] and following balanced dietary patterns, such as the Mediterranean diet and the DASH diet, which include moderate consumption of saturated fatty acids, minimal consumption of sugars and ultraprocessed products, and an increased consumption of fiber-rich foods, contribute to a favorable environment for gut microbiota, preventing metabolic endotoxemia, insulin resistance and reducing the risk for the development of cardiometabolic diseases [109]. In addition, higher adherence scores to these dietary patterns induce an increase in bacterial diversity [109], which favors the maintenance of a stable gut microbiota.

## Factors that influence food intake

Food intake is determined by food choices [110], which are widely varied and influenced by intrinsic and extrinsic factors [111] that interact in a complex and changing manner, influencing the development and maintenance of these choices [112, 113].

### Intrinsic determinants

The desire to eat is influenced by the metabolic demands and the human body has several mechanisms that regulate food intake in order to maintain an adequate energy balance [114, 115]. Appetite control is complex and involves the integration between neural circuits including the hypothalamus (homeostatic control), the mesolimbic system (hedonic control) and the frontal lobe (executive control). The cross-talk between homeostatic and hedonic factors is mediated mainly by the adipose tissue, the pancreas and the intestine [2, 116–119]. These mechanisms frequently become dysregulated and lead to excessive food intake, which may lead to weight gain [120–122].

The hedonic and reward processes are important motivators for decisions and are strong enough to counteract the homeostatic needs. Food selection and intake in humans are largely driven by the interaction between homeostatic control and reward signals. This interaction requires the complex involvement of superior cognitive functions, including memory, learning, and assessment of different options [123–125].

People can use food and nutrition knowledge to change their behavior, but knowledge itself is unlikely to be effective [112], not being enough to achieve substantial and long-term behavior change [126]. It was demonstrated in a study that evaluated fast food consumption, in which the participants reported to be more influenced by convenience, satisfaction, family, friends, and other facilitating factors [127].

### Extrinsic determinants

The eating habits are also shaped by the environment in which the individual is inserted, with decisions about food choices being situational and shaped by the physical (urban life vs. rural life or the lack of access to healthy foods in poor regions), economic, social and cultural environment [128, 129].

Dietary changes require the acquisition of new knowledge and skills, and the time and discipline needed for food preparation. In the Brazilian population, choices are strongly linked to socioeconomic factors [130]. Even though an increased income does not necessarily result in improvements in eating quality, the cost clearly influences food choices [131]. Food access and availability also have a major impact on decisions regarding food. For instance, foods like fish, lean meats, and fresh fruits and vegetables might be less available in poorer rural and urban communities [128, 132].

On the other hand, ultraprocessed and high energy density foods are readily available in a broad range of products and a diet with a high proportion of these foods increase calorie intake, which might lead to weight gain [133] and increase the risk of developing chronic diseases [134].

Another factor is food architecture [135–137], in which food placement, design, and the characteristics of the environment where food is consumed can positively or negatively influence food choices [138–144].

The exposure to different diet compositions and eating habits in the first years of life occur in the home environment, where the family can provide reference standards to assess and judge eating behaviors as “correct”, “normal”, “inadequate” or “acceptable”, that will be used throughout life [145–149].

Together, environmental conditions and social experiences exert a continuous impact on eating behavior [150, 151] Most part of the meals happen in the presence of other people [146] and the decisions regarding food choices are not only individual, but also influenced by the group [152].

An additional important factor for food intake is the cultural standard, including the norms learned throughout life and that can be shared with a group of people. For example, the composition of specific meals can vary according to cultural reasons, since not everything available to be eaten is culturally accepted [153].

Nowadays, the media, especially social media, is the main source of information about food and nutrition for many people and can also influence food intake [154]. High exposure to content related to a perfect body image has a negative impact on the treatment, due to the frustration of not being able to achieve this perfection. Also, because young adults are vulnerable to the influence of social media, it can impact the choice of healthy foods [155, 156].

There is also a growing argument about the influence of sustainability as a determinant of food intake, through the choice of a diet that promotes a sustainable food system and limits carbon emissions, such as the reduction of the consumption of ultraprocessed foods and beverages and the adequacy of red meat and dairy intake [157–159].

In summary, it is important to correctly understand the conscious and unconscious factors that influence food choices. The process of change must be gradual and promote sustainable habits.

**Table:** 

Statement	Class of recommendation	Level of evidence
1. Nutrition education strategies for individuals that are willing to change dietary intake is recommended	I	A
2. The provision of sustainable strategies for moderating energy intake should emphasize diet quality while maintaining palatability	II	B
3. Nutritional follow-up should be associated with behavior change strategies	I	B

## Nutritional assessment

Nutritional assessment is essential to determine the nutritional diagnosis of the individual and to outline strategies for the prevention of obesity [160]. It should include the assessment of food intake, social history, socioeconomic condition, motivation for weight management, history related to food and nutrition, home environment, eating behaviors, presence of food allergies, history of previous diets, use of medicines and supplements, and physical activity practice [160], which might assist in providing a tailored weight-control program.

The assessment also includes anthropometric measurements considering body composition, weight history, and current weight, height, and BMI [160]. The determination of the BMI (calculated in kg/m^2^) is a simple and low-cost method to assess the nutritional diagnosis. The measurement of waist circumference is important to complement the diagnosis provided by the BMI, because it gives an estimation of body fat distribution and potential cardiometabolic risks [160, 161]. Moreover, biochemical data, patient clinical history and family history can allow a more individualized treatment [160].

The resting metabolic rate (RMR) should be used to determine energy requirements in adults with overweight or obesity using indirect calorimetry, when available, or using predictive equations such as Harris-Benedict [162] or Mifflin-St. Jeor [163, 164], that use actual weight to estimate RMR. From this value, the estimate of the total energy expenditure (TEE) can be done by multiplying the RMR by a physical activity factor: sedentary (≥ 1.0 < 1.4); low active (≥ 1.4 < 1.6); active (≥ 1.6 < 1.9) and very active (≥ 1.9 < 2.5) [165]. The meal plan can be based on this value to promote a negative energy balance.

Food intake assessment is essential for nutritional monitoring to prevent obesity and noncommunicable chronic diseases and as part of the treatment program to control these diseases. In this context, there is consensus in the literature that eating is one of the modifiable or controllable factors, and the assessment of food intake is the most used indirect indicator for the diagnosis of the nutritional status and, consequently, of individual and population health risk [166].

There are day-to-day variations that affect food consumption and hinder an exact assessment of food intake. For instance, there is a daily variation in food consumption (week days/weekends), during holidays and vacations, among others [166].

Some very important limitations that must be considered are the presence of omission and underreporting; they can be non-intentional and should be interpreted with caution. Underreporting can be composed by under-recording, when there is a failure to record everything that is consumed, and/or by undereating, when there is a consumption of less than usual because of the requirement to record food intake [167, 168].

Food intake assessment should not only consider the quality and amount of food eaten, but also how the individual eats; it is important to identify obstacles that prevent the patient from making permanent eating behavior changes. This information will allow for a better planning and implementation of diet therapy, which in the case of the obesity treatment will increase the chance of a good result and adherence to treatment [166].

Nutritional assessment contributes to the choice of appropriate strategies for nutritional counseling, providing advices based on scientific evidence and that contribute to eating pleasure.

## Energy density

Energy density is defined as the amount of energy in kcal or kilojoules (kJ) in a particular weight (g or 100 g) of food and beverages [169–171]. Although it does not differentiate qualitative aspects, a diet with low energy density (LED) is correlated with better overall diet quality [172–175], especially because of the higher intake of fruits and vegetables and lower consumption of saturated fats and sugars [173], mostly from sweetened beverages [174].

A meta-analysis of nine randomized clinical trials (RCTs) and four observational cohort studies (n = 3628) showed association between LED—mainly by strategies to reduce the consumption of fats and/or sugars—and moderate weight loss (− 0.53 kg) [171].

A systematic review that investigated the association between LED and changes in body weight in adults, including 17 studies (seven RCTs, one non-randomized trial and nine cohorts) [172], reported that LED was associated with weight loss, ranging from 0.05 to 7.9 kg, in the non-randomized trial and in four of the RCTs [176–180]. One of the studies was conducted in women with excess weight (n = 49) and lasted 10 weeks. In the groups that received 300 g/day of apples or pears, there was a decrease in energy density (− 1.23 and − 1.29 kcal/g, respectively) and weight reduction (− 0.93 and − 0.84 kg, respectively), compared to the group that received 60 g/day of oat cookies [178]. In another RCT in adults (n = 82), even though the consumption of high energy density snacks (> 3 kcal/g) with the meal was associated with weight gain, the consumption of LED snacks (< 1 kcal/g) together with or between meals had no influence on weight and body composition changes [181]. The other RCTs investigated showed no association between LED and variation in weight [172, 182, 183]. The evidence from prospective cohort studies, three of which had more than 48,000 participants [184–186] reported a positive association between LED and less weight gain, lower BMI [184, 187–189], improved maintenance and/or weight loss [190, 191], and lower waist circumference [172, 185, 186].

Hall et al. [133] conducted a crossover RCT in which adults (n = 20) were admitted to a clinical research center and received isocaloric meals, of the same energy density and macronutrient composition, with ultraprocessed or unprocessed foods. At the end of the study, the diet with meals composed by ultraprocessed foods resulted in increased ad libitum calorie intake (+ 508 kcal/day) and, consequently, led to weight gain (+ 0.9 kg) compared to the group that consumed unprocessed foods [133]. A RCT in adults with excess weight (n = 183) submitted to calorie-restriction intervention (1200–1500 kcal/day) observed that an increased consumption of foods with lower energy density was associated with reduction in BMI; these findings can be justified by the fact that these foods tend to have more fiber and increase dietary volume, promoting satiety [171, 192, 193] and, consequently, contribute to increased diet adherence with long-term calorie restriction [192].

Diets with low energy density might be associated with healthier eating patterns [172–175], due to increased intake of fiber, dietary volume and lower consumption of sugars and fats in excess, promoting better body weight control.

**Table:** 

Statement	Class of recommendation	Level of evidence
Consumption of foods and beverages with low energy density associated with energy deficit from diet can be useful in the treatment of obesity	IIa	B

## Energy density

Portion size represents the amount of foods or beverages served and consumed in a single occasion [194, 195]. In the last decades [196], an increase in portion size of foods and beverages was observed—especially those with high energy density—this phenomenon being associated with weight gain [197].

In a systematic review and meta-analysis of data from the Cochrane Library including 69 RCTs, published by Hollands et al. [198], there was moderate-quality evidence that the exposure to increased portions, packages or dish sizes resulted in increased food intake, with an additional intake of 189 kcal/day in the diet of children and adults [198]. A meta-analysis of 30 studies investigated the effect of manipulating portion size and reported that doubling the portion size increased consumption by 35%, on average [199].

A crossover RCT in adults (n = 23) that received 50% larger portions of caloric foods and beverages for 11 days demonstrated an increase of 423 kcal/day in energy intake, compared to the same period in which the participants received regular-size portions [200]. In a RCT with preschool children (n = 69) and schoolchildren (n = 18) randomized to receive either a small or large cereal bowl (volume size of 227 g and 456 g, respectively), the use of a larger bowl induced the preschool children to request 87% more cereal to the adults and schoolchildren consumed 52% more than when given a smaller bowl [201].

Some factors that influence the effect of portion size on food intake are described, including interindividual differences in the ability to estimate the appropriate portion size [202], the perception of healthiness of foods or beverages [203], and mechanisms such as the “unit bias”. This phenomenon suggests that the visualization of a portion, as of a sandwich or cookies, determines the amount to be eaten in a single occasion, regardless of its dimension [204]. It is proposed that such effect is related to the adequacy mechanism in which the portion—instead of internal cues of hunger or satiety—guides the amount of foods or beverages consumed [205]. Additionally, larger portions of foods and beverages are also offered at a proportionally lower cost [206] and the concept of “value for money” was identified as a relevant incentive for consumers, that would be willing to buy larger portions. Because of the lower unit price, people tend to pay more for a portion larger than needed, even when smaller portions are available [196].

Several factors might determine the influence of portion size on food consumption, and increasing portions, packages and/or dishware sizes, especially of food and beverages with high energy density, may be associated to excessive calorie intake.

**Table:** 

Statement	Class of recommendation	Level of evidence
Portion size control can help to reduce the excessive consumption of foods and beverages	IIb	B

## Breakfast

Breakfast is one of the three main meals of the day and is defined as the first meal in the morning [207].

The lifestyle of modern society has changed the dietary habits of the population. In this regard, decreased breakfast consumption is pointed as an important alteration of present-day eating behavior [208].

Regular breakfast consumption is a factor that seems to influence body weight [209].

Inadequate dietary patterns during childhood and adolescence are one of the main risk factors for the early development of obesity and other chronic diseases [210]. A cross-sectional study was conducted with data from the 2015 National Adolescent School-based Health Survey—PeNSE (n = 10,926 adolescents), with the objective to identify and assess dietary patterns among Brazilian adolescents. Two dietary patterns were identified: the first was characterized by unhealthy-eating markers (candies, fried salty snacks, soft drinks, among others); and the second by healthy-eating markers (beans, fresh fruits and vegetables). Adherence to the unhealthy pattern was positively associated with the female gender, breakfast omission, eating meals without parents and/or guardians, eating while studying or watching TV and frequenting fast food restaurants [211].

The International Study of Childhood Obesity, Lifestyle and the Environment (ISCOLE) [210] examined the association between breakfast frequency and adiposity in a sample of 6,941 children aged 9–11 years from 12 countries, between 2011 and 2013. Frequent breakfast consumption (6–7 days/week) was associated with lower BMI z-cores and lower body fat percentage compared with occasional (3–5 days/week) and rare (0–2 days/week) breakfast frequency. However, associations were not consistent across all 12 countries [210].

RCTs examined the influence of breakfast consumption on weight loss. All were short-term studies (16 weeks) and none of them reported increased weight loss among those consuming breakfast [212, 213].

There is some evidence that the type of food consumed in the breakfast can influence energy intake at the end of the day. Egg consumption at breakfast, compared to an isocaloric breakfast high in carbohydrates, promoted greater weight loss, possibly by increasing the levels of satiety hormones like glucagon-like peptide 1 (GLP-1) and peptide YY (PYY) [214, 215]. A study showed that the consumption of a breakfast with eggs did not reduce the amount of food eaten at lunch by children and adolescents [216]. On the other hand, egg consumption at breakfast reduced food intake at lunch in women with overweight, compared to a breakfast with bread [217]. The mechanism behind this effect is not fully clear, but it does not seem to be related to the high-quality protein of eggs [218].

A 12-week randomized controlled trial compared the effect of a high-protein versus a low-protein breakfast in subjects who did not routinely eat breakfast, with a control group who continued to skip breakfast. In this study, the amount of protein eaten at breakfast did not influence body fat percentage and weight gain [219].

Another 12-week randomized controlled study compared the consumption of a high-fiber, low-fat breakfast with a control group that consumed their habitual breakfast and found no effect of the high fiber content on body weight [220].

Despite conflicting results on the subject, future studies should control for factors associated with obesity that might confound the results (such as sleeping habits and circadian rhythm) and also can influence body weight and explain the associations between breakfast and obesity [221].

**Table:** 

Statement	Class of Recommendation	Level of Evidence
There is no conclusive evidence if breakfast plays a causal role both for weight gain and weight loss	IIb	A

## Fruits and vegetables

According to the VIGITEL (Surveillance System for Risk and Protective Factors for Chronic Diseases by Telephone Survey) [222], 34.3% of the Brazilian population eats fruits and vegetables on five or more days of the week. In addition, the recommended intake of at least 5 daily portions of fruits and vegetables was observed in only 22.9% of the subjects [222]. Fruits and vegetables contribute significantly to human nutrition, being a major source of micronutrients, phytochemicals, and dietary fiber [223]. Low intake of these foods is inversely associated with an increased risk of noncommunicable chronic diseases, including cardiovascular diseases (CVDs) and certain types of cancer [197, 223–225]. The investigation by Wang et al. [226] with data from the Nurses' Health Study (NHS) (n = 66,719) and the Health Professionals Follow-up Study (n = 42,016) showed that the intake of 5 portions of fruits and vegetables was associated with lower mortality. Furthermore, a meta-analysis including these two cohorts and the results of other 24 prospective cohorts (n = 1,892,885) showed that higher intake of fruits and vegetables, with the exception of fruit juices and starchy vegetables, was associated with lower mortality [226].

In a systematic review and meta-analysis of 17 cohort studies (n = 563,277), higher intake of fruits was associated with modest weight loss (− 13.7 g/year per 100-g increment) and decreased waist circumference (− 0.04 cm/year per 100-g increment) [227]. In parallel, in a systematic review of 23 publications, increased intake of fruits and vegetables concomitant with decreased intake of high energy density foods led to lower adiposity in adults [228]. A RCT in adults with excess weight (n = 125) showed that the replacement of foods rich in fat and energy with at least 400 g/day of vegetables and 300 g/day of fruits resulted in greater weight loss compared to the control group [229].

In a review of 27 cohort studies and RCTs, higher intake of fruits and vegetables was associated with less weight gain or regain in pre and post-menopausal women [230]. In the analysis of the NHS II, a prospective cohort with 73,737 participants, the authors observed, after 4 years, a discrete change in body weight of − 0.27 kg per daily serving of fruits and of − 0.16 kg per daily serving of vegetables [231].

Fruits and vegetables figure as a significant dietary source of micronutrients, phytochemicals and fiber, contributing to decreased risk of developing noncommunicable chronic diseases and, associated with reduce caloric intake, promotes weight loss.

**Table:** 

Statement	Class of recommendation	Level of evidence
Encouraging the intake of fresh fruits and vegetables, associated with a hypocaloric diet, contributes to weight management	IIa	A

## Fast-food

The term fast food refers to convenience meals prepared out of the home, planned to be readily available for consumption, being usually produced by a highly mechanized process [232]. Frequently, typical fast food meals are served in large portions, characterized by an unfavorable nutritional profile with excess saturated and/or trans fat, sugars, and sodium [195, 233, 234].

In particular, an expansion of fast food restaurant chains that sell unhealthy foods is observed in Latin America [235] and the increased consumption contributes to excessive calorie intake and, consequently, increased risk of obesity [163, 233]. The urbanization and economic growth processes of low and medium income countries have promoted changes in eating patterns, encouraging the intake of foods with high energy density and poor nutritional quality [236, 237]. Among the determinants of fast food consumption is the proximity and density of this type of establishment in the surroundings, even though the influence results mainly from the interaction between economic and sociocultural factors [234].

A cross-sectional study investigated the influence of fast food consumption on body weight in subjects (n = 270) in the preoperative period of bariatric surgery. Weekly frequency of fast food consumption was 2.68 and for each unit increase in fast consumption there was a 26% greater risk of presenting BMI ≥ 50.0 kg/m^2^ versus BMI between 30.0 and 39.9 kg/m^2^ [233]. In a prospective cohort with 10,162 adults followed by approximately 4.6 years, the subjects who reported frequent snacking habits presented higher fast food intake and had a 66% greater risk of gaining ≥ 3 kg of body weight per year compared to peers who did not present this behavior [238].

Even though there is a need for more researches for elucidation regarding strategies to promote change in food systems and food services, initiatives to reformulate fast food meals, aiming to improve nutritional quality [239], ensure transparency of nutrition information and the ingredients of the commercialized products [195, 239], promote consumer awareness about portion size [233], as well as regulations to set proportional prices for foods and beverages in order to create incentives to prevent excessive portion sizes [235, 239] might be relevant to mitigate the risk of excess weight.

Eating patterns characterized by high consumption of fast-food meals, typically presenting high energy density and poor nutritional quality, contributes to excessive calorie intake and, consequently, increased risk of obesity.

**Table:** 

Statement	Class of recommendation	Level of evidence
The intake of fast-food meals high in sugars, sodium, and saturated and/or trans fat is not recommended	IIb	C

## Ultraprocessed food

The term “ultraprocessed food” was originally presented in the Dietary Guidelines for the Brazilian Population 2014 [224], and was further adopted by researchers from different countries including the United States [240], European countries [241, 242], Canada [243] and Australia [244], among others, being widely used in studies about food consumption and excess weight. Ultraprocessed foods are those with ingredients which are not used in culinary preparations (corn syrup, hydrogenated or interesterified fats and other substances used to increase palatability or make the product more attractive, including flavor enhancers, emulsifiers, bulkers, and coloring agents) [245]. Furthermore, it is important to mention that these products are prepared with high amounts of sugars and fats, a combination that is not present in natural unprocessed foods, and can also include high amounts of salt.

An important randomized controlled crossover clinical trial showed that ultraprocessed foods cause excess calorie intake (an additional of 508 kcal/day) and weight gain [133].

The French NutriNet-Santé cohort (n = 110,260) and a study conducted in Spain (n = 8451) showed that the increased intake of ultraprocessed foods is associated with higher BMI and increased risk for the development of overweight and obesity [246, 247]. A recent systematic review and meta-analysis with data from 891,723 participants revealed that the intake of ultraprocessed foods was associated with increased risk of overweight, obesity, abdominal obesity, metabolic syndrome, and all-cause mortality in adults and also with metabolic syndrome and dyslipidemia in children [134].

**Table:** 

Statement	Class of recommendation	Level of evidence
The consumption of ultraprocessed foods is associated with excessive calorie intake and weight gain	III	B

## Sweetened beverages

Sugar-sweetened beverages contribute to a positive energy balance and are known for its strong association with weight gain, contributing to the global obesity epidemic [248, 249], representing a serious public health problem [250]. According to data from the VIGITEL, the intake of soft drinks was 16% lower in 2019 compared with 2006 [222]. In contrast, the per capita intake of nectar juices virtually doubled in 2019 compared to 2010 [251]. Juice contains 100% fruit pulp in its composition, while nectars and refreshments contain between 20 and 40% [252, 253].

Excessive fructose intake both from sucrose or corn syrup increase plasma levels of triglycerides by the activation of hepatic lipogenic pathways via the Carbohydrate Responsive Element Binding Protein (ChREBP), a transcription factor that promotes the synthesis of hepatic lipogenic enzymes involved in the synthesis of fatty acids [254]. Fructose present in sugars induces a faster synthesis of fatty acids compared to glucose, because of the lack of a negative feedback mechanism in the liver [255–257]. Furthermore, excess fructose leads to increased synthesis of hepatic glucose, insulin resistance and higher adipose tissue inflammation [258].

Alcoholic beverages also increase the risk of T2DM [259, 260]. However, habitual intake of sugary beverages was likewise associated with higher incidence of T2DM, regardless of the degree of adiposity [261]. In addition, contribute to increased cardiovascular disease risk [262–268].

The systematic reviews and meta-analyses from the Nutrition and Chronic Diseases Expert Group (NutriCoDE) study [269] demonstrated that sugar-sweetened beverages increase the risk of CVDs and T2DM and the BMI [269]. The analysis of data from the prospective cohorts NHS (1986–2012), NHS II (1991–2013) and Health Professionals’ Follow-up Study (1986–2012) showed that sugar-sweetened beverage intake was associated with a 16% higher T2DM risk [260]. The effect of the intake of sugary beverages and 100% fruit juices was assessed in 13,440 subjects from the REasons for Geographic and Racial Differences in Stroke (REGARDS) study [270], showing that among high sugary beverage consumers (≥ 10% of TEI), including unsweetened fruit juices, versus low consumers (< 5% of TEI), the risk of all-cause mortality was higher, even after adjustments for BMI [270]. A systematic review and meta-analysis of fifteen cohort studies with 1,211,470 participants showed that an additional 250 mL of sugar-sweetened or artificially-sweetened beverages resulted in increased total and cardiovascular mortality [271].

A randomized crossover blind trial conducted in Brazil assessed the effect of the consumption of sugar-sweetened beverages in 17 adults who practiced regular physical activity and had an average BMI of 24.59 ± 3.98 kg/m^2^ [272]. The total volume of the drink was 1.5L, taken four times during the day. The consumption of sugar led to increased weight, waist circumference and plasma levels of triglycerides. Besides that, it decreased physical performance and the cardiovascular response during exercise [272].

An important study with data from 71,935 participants (aged > 60 years) from the NHS assessed frailty (defined as the presence of three of the five criteria from the frailty scale: fatigue, poor strength, reduced aerobic capacity, having ≥ 5 chronic illnesses, and weight loss ≥ 5%) [273]. The presence of frailty was assessed every four years from 1992 to 2014, and the association with the consumption of sweetened beverages was investigated. After adjustment for diet quality, BMI, smoking status, and medication use, consumption of ≥ 2 servings/day of sugar-sweetened and artificially-sweetened beverages was associated with higher risk of frailty versus no consumption.

The Dietary Guidelines for the Brazilian Population [224] and the 2020–2025 Dietary Guidelines for Americans [195] recommend limiting the intake of sugar, sugar-sweetened beverages and fruit juices, even unsweetened. Fruit juices not always offer the same benefits of whole fruits, which are important sources of fiber and other essential nutrients.

Sugar-sweetened beverages represent a significant source of sugar in the diet, contributing to positive energy balance and weight gain, in addition to higher risk of CVDs and T2DM development.

**Table:** 

Statement	Class of recommendation	Level of evidence
Sweetened beverages or even unsweetened fruit juices contribute to increased calorie intake and body weight, and should be discouraged for the prevention and treatment of obesity	I	A

## Sweeteners

Sweeteners are classified as nutritive or non-nutritive [274, 275], contain very little calories or are calorie-free [275]. The regulation and safety assessment of food additives such as sweeteners is determined by the Joint FAO/WHO Expert Committee on Food Additives (JECFA) [276]. This committee determines the acceptable daily intake (ADI) for each additive [276, 277].

The consumption of sweeteners has increased in recent years and beverages have been its main source [278]. Intake is higher among individuals with obesity and increases with age [278]. However, an important study conducted in Chile revealed that children can exceed the safe levels of sweeteners consumption by consuming large amounts of artificially sweetened foods [279].

A double-blind study [280] conducted in normal-weight men tested the acute effects of beverages sweetened with stevia or aspartame or a beverage sweetened with 65 g of sucrose, and showed that the artificially sweetened beverages induced higher scores for the desire to eat and hunger and a lower feeling of fullness. Some studies demonstrated the benefit of using sweeteners [275, 281, 282], while others showed association with weight gain [283] and increased risk of T2DM [284].

A randomized clinical study compared the effect of sweetener consumption on weight loss in 303 individuals with obesity or overweight [285]. All participants were included in a weight loss program and instructed to consume 710 mL of water or artificially sweetened beverages for one year (12 weeks of weight loss followed by 40 weeks of maintenance). Sweetener consumption led to greater weight loss (− 6.21 kg ± 7.65 kg), accompanied by a decrease in waist circumference, compared to water intake (− 3.45 ± 5.59 kg). Therefore, the authors suggest that the inclusion of artificially sweetened beverages in the meal plan contributes to the treatment of obesity. As limitations to the study, they mention the fact that only individuals who already habitually used sweeteners were included [285]. However, a systematic review and meta-analysis with data from MEDLINE, Embase, and Cochrane, conducted with seven randomized controlled trials with a follow-up of up to 6 months (1003 participants) and thirty prospective studies with a follow-up of 10 years (405,907 participants), do not support the same benefits attributed to the use of artificial sweeteners [286]. Evidence from prospective population studies showed that the consumption of sweeteners was associated with an increase in body weight and waist circumference, in addition to a higher incidence of hypertension, T2DM, metabolic syndrome, and cardiovascular events [286].

In another systematic review, 57 observational and clinical studies were evaluated for qualitative and quantitative analysis of the effect of sweeteners [287] on body weight. Healthy adults or children with or without overweight or obesity were included. In general, studies have not shown an association between sweetener consumption and body weight or eating behavior [287].

The analysis of data from three large prospective cohort studies conducted in the United States observed an increase of 16–18% in the risk of developing T2DM with the consumption of artificially sweetened beverages [288]. The analyses were adjusted for the participants' BMI and similar results were observed with the consumption of whole-fruit juices or those sweetened with sugar [260]. A systematic review with meta-analysis including data from fifteen cohort studies showed a positive association between the consumption of artificial sweeteners and all-cause mortality and mortality from cardiovascular causes, both with a linear dose–response relationship [271].

A study conducted in Brazil with 1,323 adult individuals who answered a questionnaire with questions about the consumption of sweeteners and “diet” foods and anthropometric and health characteristics, showed that 53.3% of the participants use these products and that consumption increases with age [289]. Also, having difficulty to manage weight increased the likelihood of consuming these products by 2 to 3 times. The difficulty in maintaining body weight was reported by 64.8% of sweeteners consumers and the main justification for its use was the saving of calories, to be consumed in other foods [289].

In several guidelines on nutritional recommendations there is no indication for the use of sweeteners as a strategy for the treatment of obesity. In the Guideline Recommendations for Obesity Management, a document prepared by researchers at Johns Hopkins University, sweeteners are not listed in the fifteen recommended nutritional approaches [290]. The Canadian Guidelines: Medical Nutrition Therapy in Obesity Management [288] discusses that it is possible that the use of sweeteners may contribute to the treatment of obesity, but this is not confirmed in RCTs. The Dietary Guidelines for Americans (2020–2025), developed for the general population, do not include the use of sweeteners as part of a healthy eating pattern [195]. The American Diabetes Association [291], on the other hand, indicates that sweeteners can help to reduce overall calorie and carbohydrate intake, in general, when they replace sugar, but as long as individuals are not compensating with additional calories from other foods. Individuals should be advised to decrease both sweetened and nonnutritive-sweetened beverages. They suggest the importance of indicating other alternatives, emphasizing a proper hydration through water intake [291].

Current evidence from studies related to the use of sweeteners and obesity are divergent. Sweeteners can be used in the treatment of individuals with obesity, but it must be part of a balanced diet that is part of the lifestyle change.

**Table:** 

Statement	Class of recommendation	Level of evidence
There is inconclusive evidence about the effect on weight loss of sweeteners when used alone, without healthy food choices accompanied by an energy deficit	IIb	B

## Low-calorie diets (LCDs)

The identification of effective strategies for long-term weight management is crucial for the reduction of the alarming global prevalence of overweight and obesity. The treatment of obesity using different forms of nutrition intervention requires the achievement of negative energy balance through a decreased energy intake [292]. The proportion of macronutrients in the diet (fat, carbohydrate and protein) relative to the TEI has received substantial attention in the last decades because of its potential relevance for weight loss and maintenance [292, 293]. While some studies focused on isolating or eliminating specific food groups and/or nutrients, recent evidence has shown that poor diet quality and excessive amount of food are the drivers of energy imbalance and, therefore, of obesity [294].

It is well established in the literature that a calorie-restricted diet for weight loss is the first line treatment for individuals with overweight and obesity [295]. According to the Academy of Nutrition and Dietetics [163], LCDs have been traditionally defined as a diet with a balanced proportion of protein, carbohydrate and fat in reduced amounts, to provide an energy intake usually > 800 kcal/day and typically ranging from 1200 to 1500 kcal/day for women and from 1500 to 1800 kcal/day for men [163, 296, 297]. LCDs are usually planned to promote an energy deficit of 500–750 kcal/day relative to the TEE and are recommended in association with behavior change [160, 297].

The structure of the LCD can be divided in two categories: prescription of a conventional LCD meal plan, in which every food choice and portion size for all meals and snacks are defined [163, 298, 299]; and as part of a meal plan that includes one or two portion controlled meal replacements (MR) associated with a LCD including conventional meals and snacks [300].

The methods used with the aim of elaborating a structured diet, with an organized meal plan, are considered very useful to increase the adherence to the LCD, since they are convenient and reduce the need to make food choices, which can be difficult for some individuals [6]. Besides that, MR can increase diet adherence providing controlled portions and greater convenience [296, 301, 302].

Although there are publications showing positive results of calorie-restricted diets on weight loss in the short term, in clinical practice it is frequent that doctors, dietitians and patients are disappointed with the long-term results, which reveal a high frequency of weight regain [298, 299].

A review that examined the results of 16 studies on the effect of LCDs on weight loss showed that in the studies with a minimum follow-up of 3 years (n = 6163), there was an average loss of 3.5% of body weight, and with 4 years of follow-up (n = 5696) there was an average loss of 4.5% of body weight compared to the control group. Most of the study participants regained weight and the dropout was high [294]. The authors mention as limitations of this review that fact that few studies reported outcomes related to weight loss maintenance, besides the high heterogeneity among the studies included, such as absence of control groups and high dropout rates [294].

The high frequency of weight regain observed after a calorie-restricted diet is partially explained by the reduced metabolic adaptation, as demonstrated in a study that compared individuals that reached the weight loss goal compared to another group that did not reach the goal [303]. In this study, the participants were submitted to a LCD (900 to 1000 kcal). Despite the similar increase in energy expenditure and fat oxidation in both groups, the participants who reached the weight loss goal presented a reduced metabolic adaptation (− 80 kcal) compared to those who did not reach the goal (− 175 kcal) [303].

Considering these findings about the results of LCDs, it is important to consider the concurrent use of other interventions that foster lifestyle changes. In this sense, relevant studies, such as the Diabetes Prevention Program (DPP) and the Look Ahead study were conducted to promote lifestyle change.

The DPP is a RCT with a duration of 3 years, in which the incidence of diabetes in high-risk adult patients was reduced in 58% with an intensive lifestyle intervention (ILI) and in 31% with the use of metformin, compared to the placebo group [304]. The Diabetes Prevention Program Outcomes Study (DPPOS) followed the participants from the DPP to investigate the persistence of these effects in the long term. In the 10-year follow-up (since the randomization for the DPP trial), the original lifestyle change group lost and then partially regained weight, reaching the end of the study with a weight loss of 2 kg. The modest weight loss reached with metformin was maintained. Diabetes incidence during the study was 4.8 cases per 100 person-years in the ILI group, 7.8 in the metformin group, and 11 in the placebo group. Despite of the weight regain observed during the 10-year follow-up, diabetes incidence was reduced in 34% in the lifestyle intervention group compared to 18% in the metformin group [304].

The Look Ahead study followed 5145 individuals with diabetes and overweight or obesity for 8 years [305]. The study had two arms: the first was an intensive program consisting of frequent meetings, individual nutrition counseling and exercise program, and the second was a less intensive program with counseling meetings about diet and exercise every four months during the first year, and annually thereafter. After 8 years of follow-up, 36% of the participants in the less intensive arm and 50% of the participants in the intensive arm achieved 5% weight loss. Weight gain above baseline occurred in 39% of the participants in the less intensive arm and in 26% of the participants in the intensive arm. Overall, weight loss was 2.6% greater in the intensively treated group, and the dropout rate was 6%. The Look Ahead study shows that the rate of weight regain is very high between 2 and 3 years after the beginning of the weight-loss intervention. After 3 years, even though there is still some weight regain, the rate becomes stable and an estimate of the long-term effect on weight can de done [305].

Another study reports that 122 individuals with overweight and obesity with BMI between 25 and 34 kg/m^2^, aged between 30 and 59 years, adhered to a 3-year intervention with the aim of achieving a 5% reduction of the initial weight [306]. During the study period, the participants were subjected to moderate caloric restriction, with an approximate energy deficit of 100 kcal/day. Food intake was assessed using a semi-quantitative food frequency questionnaire and a 24-h dietary recall. Adequate weight loss was defined as a reduction of 2 kg from the initial weight after the clinical intervention period. Fifty individuals lost 2 or more kg and were included in the small weight-loss group, and 49 individuals lost less than 2 kg and were included in the unsuccessful group. In the long term, the first group presented lower levels of inflammatory cytokines and less inflammation-induced oxidative stress in individuals with overweight and obesity [306].

A long duration RCT (4 years) subjected 419 postmenopausal women with overweight or obesity to a lifestyle change intervention or to a Health Education group (control) [307]. The intervention group followed a healthy eating pattern with specific recommendations, such as: (1) reduce the intake of sweets, sweetened beverages, total fat, saturated and trans fats; (2) reduce caloric intake; (3) increase the consumption of foods rich in soluble fiber; (4) increase the consumption of fruits, vegetables, and whole grains. Changes in eating pattern were positively associated with weight loss. The authors observed that the decrease in the consumption of desserts and sweetened beverages was associated with weight loss both in the short (6 months) and in the long term (48 months) and with weight maintenance, while the adoption of the habit of eating fruits and vegetables, together with a reduced intake of meat and cheese, were additional factors that contributed to long-term weight loss [307].

A meta-analysis of six randomized trials compared weight loss after a LCD using conventional foods or with MR for individuals with overweight or obesity. The intake of prescribed calories was the same for both groups. The group that received meal replacements lost more weight at 3 months and 1 year (− 2.54 kg and − 2.43 kg, respectively) than the group that received the conventional diet [296].

A systematic review aiming to elucidate the efficacy of LCDs to reduce the hepatic volume in patients waiting for bariatric surgery included eight studies (n = 251) that used nine LCDs with different dietetic characteristics (800–1200 kcal, for 2 to 8 weeks). The LCD reduced hepatic volume (12–27%) and weight (4–17%), particularly during the first weeks. Based on these findings, the LCD was considered more efficient than very low-calorie diets (VLCDs, 450–800 kcal) for 2 to 4 weeks in the preoperative period of bariatric surgery [308].

Even though adiponectin is secreted by the adipose tissue, its serum concentrations are inversely correlated to body fat mass and obesity [309]. There is evidence showing increased adiponectin levels in patients following a LCD, with the intake of polyunsaturated fatty acids, fish oil, protein, and the adherence to balanced diets, including the Mediterranean Diet [309].

A systematic review and meta-analysis of 13 clinical trials examined the effect of LCDs on adiponectin levels compared to control groups without calorie restriction [310]. The study showed that the weight loss diet results in increased adiponectin levels. This effect was greater in short-term studies (≤ 16 weeks) compared to long-term studies (> 16 weeks). This result can be explained by the increased adherence to the prescribed diet in the short term [310].

Results from RCTs and systematic reviews reinforce the effectiveness of LCDs for the treatment of obesity. However, to ensure long-term success, lifestyle changes are required in the process.

**Table:** 

Statement	Class of recommendation	Level of evidence
Low-calorie diets are fundamental for obesity treatment and the management of the meal plan must be associated with lifestyle changes	I	A

## VERY low-calorie diets (VLCDs)

Even though VLCDs are used in some clinical situations that require rapid weight loss, they are not routinely recommended for obesity treatment [311]. For instance, the American Heart Association/American College of Cardiology/The Obesity Society guidelines recommend that VLCDs are used only in limited circumstances and always with medical and nutritional support [160].

In the United Kingdom, the 115 guidelines by the Scottish Intercollegiate Guidelines Network [312] state that long-term weight maintenance after VLCDs is not superior to other options for the treatment of obesity, while the National Institute for Health and Clinical Excellence [311] guidelines recommend that these diets are reserved for individuals with BMI ≥ 30 kg/m [160, 163, 313] that present a clinical condition that require rapid weight loss, such as the placement of articular prosthesis, fertility treatment [311], and individuals with severe obesity candidate to bariatric surgery, to reduce surgery risk [163, 313].

These diets are called VLCDs because they provide less than 800 kcal/day with a high protein amount, typically from 70 to 100 g of protein/day or 0.8 to 1.5 g of protein/kg of ideal body weight/day [163] and a low amount of carbohydrate, to promote weight loss with minimum loss of lean mass and supplementing with vitamins, minerals, electrolytes, and essential fatty acids to ensure an adequate nutrition [314]. They usually involve partial or total replacement of meals and snacks with complete pre-packaged MR such as shakes, soups or bars [313, 314], depending on the availability of these products in the country.

A systematic review investigated the use of VLCDs for weight loss [7] and concluded that they are effective in the short term, but not in the long term (1 year or more) [315].

A meta-analysis of six RCTs that compared the long-term effectiveness of LCDs and VLCDs for weight loss showed that, although VLCDs result in significantly greater weight loss in the short term (4 months), 16.1 ± 1.6% vs. 9.7 ± 2.4% of the initial weight, there was no difference between the two diets in the long-term follow-up (> 1 year) [313].

A systematic review and meta-analysis of RCTs assessed the clinical effectiveness and safety of the use of VLCDs for weight loss. The study included adults (≥ 18 years old) with overweight (BMI ≥ 25 kg/m^2^) or obesity (BMI ≥ 30 kg/m^2^), with or without comorbidities. The interventions proposed in the studies involved three comparisons: VLCD vs. behavioral program; VLCD + behavioral program vs. brief intervention; and VLCD + behavioral program vs. behavioral program alone. The authors concluded that most of the studies showed that the association of VLCDs with a behavioral program led to greater weight loss at medium and long term compared to behavioral programs alone [316].

A randomized trial with 117 individuals aged ≥ 65 years with BMI equal to or greater than 32 kg/m^2^ was conducted for 12 weeks and consisted of 3 sessions/week of physical activity combined with healthy eating advice (Ex/HE), hypocaloric diet (Ex/diet), or VLCD (Ex/VLCD). The study outcomes were functional and physical abilities, body composition and nutritional parameters (albumin, vitamins B12 and D, ferritin, and folate). After 12 weeks, the percentage of weight loss was 3.7%; 5.1%, and 11.1% in the Ex/HE, Ex/diet, and Ex/VLCD groups, respectively. The Ex/VLCD group presented a significant reduction of body fat (16.8%), lean mass (4.8%), and bone mineral density (1.2%), but increased relative lean mass (3.8%). Improvements in nutritional parameters were seen in Ex/VLCD, but not in Ex/HE or Ex/diet. The authors concluded that VLCD can be of particular benefit in older adults who suffer from the immediate impact of obesity on their physical and functional abilities [317].

since VLCDs should be supplemented with vitamins, minerals, electrolytes, and essential fatty acids to ensure an adequate nutrition and are effective in the short term, they must be recommended only in limited circumstances and always with medical and nutritional support.

**Table:** 

Statement	Class of recommendation	Level of evidence
VLCDs should not be the first option for obesity treatment	IIa	A

## Dietary patterns and obesity

Dietary patterns can be defined as the amount, proportion, variety or the combination of different foods and beverages in the diet, and the frequency with which they are habitually consumed [318]. This concept is attributed to the fact that people do not eat nutrients in isolation, but a variety of components that comprise the diet. It is well stablished in the literature that dietary patterns that combine varied food groups are more efficient for the prevention and treatment of diseases such as obesity, T2DM, and cardiovascular disease. The most well-known and studied are the Mediterranean diet [319] and the DASH [320]. Therefore, international guidelines reinforce this concept and state that nutritional recommendations should not be based only on the percentage energy from macronutrients (carbohydrates, protein and fat), but on the food matrix of the meal. Despite some individual particularities, dietary patterns present common features including foods rich in fiber (fruits, vegetables, and whole grains), lean meats, low-fat dairy products and a low consumption of ultraprocessed foods high in sugar, saturated fats, and trans fat [319, 320].

In this sense, adherence scores were developed to assess the adherence to healthy dietary patterns such as the Mediterranean diet [321] and the DASH diet [322]. In the United States, the HEI-15, elaborated with 13 components that reflect adherence to the US Dietary Guidelines [323] is used. The standardization of quality indexes is widely used in prospective studies to investigate the relationship between diet quality and risk factors for certain non-communicable chronic diseases [109].

The benefits and effectiveness of the Mediterranean diet became recognized after the 1960’s, with evidence showing decreased risk of cardiovascular mortality among populations that followed this dietary pattern [324]. From these findings, this pattern was exhaustively studied in clinical and epidemiological studies with the aim of assessing its association with the prevention and treatment of cardiometabolic diseases. In particular, the Mediterranean diet also includes that consumption of nuts (walnuts, nuts, and almonds) and olive oil (OO) [321].

Another dietary pattern internationally recognized is the DASH diet, that was originally developed as a non-pharmacological therapeutic strategy to reduce arterial blood pressure [24]. This diet presents particular characteristics, including a lower percentage of fat and limited sodium intake. The preliminary study was conducted with 412 individuals with hypertension that were randomized in three groups with diets with high, moderate or low amounts of sodium [24]. The first group was taken as the control group and the second and the third groups were advised to follow a DASH diet. The greater benefit for blood pressure reduction was obtained with the combination of reduced sodium intake and DASH diet, with high vegetable intake [24]. The efficiency of this dietary pattern for the prevention and treatment of cardiometabolic diseases was tested in several clinical and epidemiological studies.

In the subsequent years, the DASH diet was also tested with regard to other cardiometabolic outcomes, and a systematic review with meta-analysis using Grading of Recommendation Assessment, Development and Evaluation (GRADE), showed that this dietary pattern was associated with reduced incidence of cardiovascular disease, stroke, hypertension, T2DM and obesity [325].

A study conducted in Brazil with a representative sample of adolescents had the objective to assess the association between a DASH diet score and the prevalence of obesity and overweight in this population [326]. The study sample comprised 71,533 adolescents from the database of the Study of Cardiovascular Risks in Adolescents (ERICA) [327]. The higher prevalence of overweight/obesity was found in boys aged between 12 and 14 years (28.2%). Among the most frequently consumed foods, sweetened beverages, red meats and processed meats stood out. The study did not show association between the DASH diet score and body weight. The authors attributed this lack of association to the fact that the adolescents that presented high intake of foods that characterize the DASH diet also consumed high amounts of non-healthy foods. Therefore, the adherence to the DASH diet was not able to outweigh the detrimental effect of the high consumption of foods rich in sugar and saturated fatty acids [326].

Another study with 2,459 adolescents (12 to 21 years old) selected from the database of the (NHANES) compared the Healthy Eating Index 2010 (AHEI-2010) dietary pattern to the DASH diet and showed an inverse association between high adherence scores to these dietary patterns and the presence of metabolic syndrome [328]. A cross-sectional study with 341 children and adolescents (6 to 13 years old) with excess weight, 62% presenting cardiometabolic risk factors and 43% with insulin resistance, also assessed the association between adherence to DASH diet and these outcomes [329]. The participants with higher adherence scores presented lower intake of calories and total fat and lower fasting insulin compared to those with the lowest DASH diet adherence score. In addition, there was in inverse association between DASH diet adherence and insulin resistance. Therefore, the authors concluded that DASH diet contributes to a favorable metabolic profile in individuals with excess weight [329].

A calorie-restricted DASH diet was tested in a 12-week controlled study with 28 sedentary elderly individuals with BMI of 32 ± 6.9 kg/m^2^ and waist circumference of 101 cm ± 16.4 [330]. The participants were divided in two groups, differing only in the amount of red meat intake, of 85 g or 170 g, respectively. At the end of the study, although there was no difference in body weight, the DASH diet led to decreased waist circumference associated with increased insulin sensitivity, regardless of the amount of red meat consumed [330].

Another crossover intervention study was conducted with 31 individuals with T2DM, randomized to a DASH diet intervention or to a control group, each for 8 weeks [331]. Both groups followed a 2100-kcal diet, that in the DASH dietary pattern was based on low energy–density foods. There was a 4-week washout period between each diet. The DASH diet resulted in significant weight loss and decreased waist circumference, as well as lower fasting glucose and glycated hemoglobin [331].

The Mediterranean diet is the most studied dietary pattern with regard to the prevention and treatment of chronic diseases, and has been cited in the most important guidelines for obesity treatment, including the 2013 AHA/ACC/TOS Guideline for the Management of Overweight and Obesity in Adults [160] and the Obesity Management for the Treatment of Type 2 Diabetes: Standards of Medical Care in Diabetes—2021, elaborated by the American Diabetes Association [297].

A cross-sectional study with people from the Gulf region assessed the Mediterranean diet score in 961 adults aged from 20 to 55 years [332]. A 14-point validated questionnaire was used, and the average adherence score was of 5.9 ± 2.03. After adjusting for confounding factors, there was an inverse relationship between the Mediterranean diet adherence score and the participants’ BMI [332].

One of the most robust intervention studies that evaluated the effect of the Mediterranean diet on obesity was conducted with 322 individuals with obesity with an average BMI of 31 kg/m^2^, divided in three groups: low-fat diet (< 30% of energy from fat), Mediterranean diet (< 35% of energy from fat)—both groups with caloric restriction, and a third group advised to follow a low-carbohydrate (low-carb) diet rich in fat (Atkins-style), with < 130 g of carbohydrates [18]. The average rate of adherence was 95.4% in the first year, and 84.6% in the second year. The Mediterranean pattern was associated with higher intake of fiber and monounsaturated fatty acids. All of the groups lost weight in the first 6 months, but weight loss was higher in participants following the low-carb diet and the Mediterranean diet compared to the low-fat diet. At the end of 2 years, weight loss was higher in the Mediterranean diet group compared to the low-carb group. This study also showed that, in the subgroup of participants with T2DM, fasting glucose levels and HOMA-IR were only reduced with the Mediterranean diet [18].

The effect of behavioral and nutrition interventions was investigated in a multicentric study conducted in 23 centers of Spain, involving 6,874 adult male participants with metabolic syndrome [333]. The participants were randomly allocated to a Mediterranean diet group or to a calorie-restricted Mediterranean diet group. The total study period was 12 months and the goals were to promote weight loss and to assess the degree of adherence to the proposed diets, using a dietary intake score. The calorie restriction intervention included monthly group meetings, motivational interviewing (MI) sessions, and frequent contact by phone. The control group only had two meetings with the team during the study. The participants were adherent to the diets and the study showed that the Mediterranean diet contributes to weight loss when associated with caloric restriction [333].

The effect of the Mediterranean diet on the plasma levels of endocannabinoids and N-acylethanolamines of individuals with risk factors for metabolic syndrome was assessed. The study included 82 individuals with overweight or obesity, with sedentary lifestyle and habitual Western diet [334]. The participants were randomized in two groups: the first consumed a Mediterranean diet tailored for their habitual energy intake and the second maintained their habitual diet. The Mediterranean diet intervention lowered plasma arachidonoylethanolamide and increased plasma oleoyl/palmitoylethanolamide, with a concomitant increase in fecal *Akkermansia muciniphila*. All of these changes occurred independent of body weight changes and were associated with the amelioration of insulin sensitivity and inflammation [334].

The Mediterranean diet provides high amounts of antioxidants, such as polyphenols including hydroxycinnamic acid, flavonoids (quercetin and catechin), resveratrol, oleuropein, and hydroxythyrosol, which exert antioxidant and anti-inflammatory actions by modulating the NF-κB pathway [335–338]. Taken collectively, the properties of the Mediterranean diet [108], involving the antioxidant activity of foods, intake of healthy fats, and high fiber intake, explain the metabolic benefits observed in individuals with excess weight.

**Table:** 

Statement	Class of recommendation	Level of evidence
The Mediterranean diet has cardiovascular benefits to the individual with obesity	I	A

## Plant-based and vegetarian diets

Plant-based diets aim to promote the intake of foods from vegetable origin and, at the same time, reduce the intake of processed foods, fats, and animal products (including meat, eggs, and dairy). It is a dietary pattern that encourages the intake of diverse vegetables (raw and cooked), fruits, whole-grains, legumes, and nuts. There is no consensus in the literature with regard to the presence of animal products in the plant-based diet. Some authors mention the inclusion of such products, particularly moderate amounts of eggs and dairy [339], while others define the plant-based diet as a strict vegetarian (vegan) diet, therefore including no animal product [340].

Vegetarian diets, in turn, are those that exclude all types of meat and meat-derived products (beef, chicken, pork, lamb, fish, seafood, among others, and products such as sausages, canned tuna, etc.), and main contain eggs and/or dairy products [341].

A review of data from observational studies and controlled clinical trials showed that vegetarian diets were associated with lower mean BMI, as well as decreased risk of cardiovascular disease, hypertension, diabetes and obesity, independent of the practice of physical activity [342].

Kahleova et al. [343] conducted a RCT to test the effect of a low-fat vegan diet and observed that thermic effect of food increased in the intervention group from baseline to 16 weeks and did not change significantly in the control group. The authors relate the increased postprandial metabolism to increased insulin sensitivity resulting from a high-carbohydrate, low-fat diet [343].

In a cross-sectional study, Montalcini et al. [344] compared the resting energy expenditure of 26 vegetarian and 26 non-vegetarian subjects matched by age, BMI and gender. The results showed a significantly higher resting energy expenditure in vegetarians than in non-vegetarians [344].

To investigate the effect of the prescription of vegetarian diets on changes in body weight, Barnard et al. [345] conducted a meta-analysis combining the results of clinical trials (of ≥ 4 weeks’ duration) and identified that the prescription of a vegetarian diet (ovolactovegetarian or vegan) was associated with a mean weight reduction of 4.6 kg. Greater weight loss was reported in studies with participants with higher baseline weights, smaller proportions of female participants, older participants, or longer durations, and in studies in which weight loss was a goal [345].

Huang et al. [346] conducted another meta-analysis of 12 RCTs (n = 1151) and also observed that subjects assigned to vegetarian diet groups lost significantly more weight than those assigned to the non-vegetarian diet groups. The possible mechanism underlying the effect of vegetarian diets on weight loss was attributed to the higher intake of whole-grains, fruits, and vegetables [346]. The authors concluded that vegetarian diets can be recommended for weight management without compromising quality of life. The analysis of data from the NHANES 1999–2004 showed that vegetarians had higher intake of fiber, vitamins A, C, E, B1, and B2, folic acid, calcium, and magnesium compared to non-vegetarians [347].

The factors that can explain the benefits of a vegetarian diet on weight management include the low energy density, higher fiber intake and the beneficial effect on gut microbiota [348–350].

However, not all plant foods have low energy density (such as beverages, sweetened juices, fried foods, and sweets), and some products targeted at vegetarian consumers can be ultraprocessed, high in saturated fat and sodium.

A RCT in New Zealand investigated the effectiveness of a community-based dietary program with the prescription of a plant-based diet with vitamin B12 supplementation for adults and elderly subjects with obesity or overweight and comorbidities (T2DM, ischemic heart disease, hypertension or hypercholesterolemia), not limiting energy intake. The program resulted in significant weight loss in the intervention group after 6 months (− 12.1 kg vs. − 1.6 kg in the control group). The participants were followed for 12 months, and the mean weight reduction was maintained in − 11.5kg [351].

The intake of vegetable protein in a plant-based diet is associated with improvements in body composition and reduced weight and insulin resistance [352]. Furthermore, in the context of a low-fat plant-based diet, the lower intake of saturated and trans fat and the relative increase in the consumption of polyunsaturated fatty acids, especially of linoleic and alpha-linolenic acids, is associated with reduced body fat mass and insulin resistance, with improved insulin secretion [353].

There seems to be an increased release of gastrointestinal hormones, such as GLP-1, and increased satiety, as observed in response to a single meal based on vegetables and tofu. However, in this study, the control group consumed a meal with processed meat and cheese, matched for energy. All participants reported increased satiety after the vegetarian meal [354].

According to the Dietary Guidelines for Americans [195], it is possible to maintain a balanced vegetarian dietary pattern, with the inclusion of vegetable protein, but there is concern about the intake of vitamin B12, which is only found animal foods. Therefore, the meal plan must include, beyond vegetables high in iron, zinc, calcium, and omega-3, as well as strategies to improve nutrient bioavailability, the supplementation of vitamin B12 for vegans, children, pregnant and lactating women, and ovolactovegetarians with irregular consumption of eggs and/or dairy [195, 355].

Vegetarian diets characterized by decreased consumption of ultraprocessed foods, ensuring adequate intake of micronutrients and leading to lower energy intake, figure as an alternative approach for weight management.

**Table:** 

Statement	Class of recommendation	Level of evidence
Vegetarian or plant-based diets with reduced intake of ultraprocessed foods can be an option for the prevention and treatment of obesity	IIa	A

## Meal replacements

MR should provide macronutrients and essential micronutrients, respecting the energy value for which they are destined (weight loss, maintenance, or gain) [356], and can be used to partially [163] or totally [160] replace daily meals. Guidelines from the American Heart Association/American College of Cardiology/The Obesity Society (2013) acknowledge the superiority of MR compared to LCDs with conventional foods in the short term, but reinforce the lack of evidence showing benefits with long term use [160]. On the other hand, the Position Statement of the Academy of Nutrition and Dietetics suggests the optional use of MR as part of the hypocaloric diet [163].

The important Look AHEAD study was designed to assess the impact of intensive lifestyle change (diet, physical activity and behavioral interventions in the ILI group) on weight and cardiovascular risk reduction in subjects with excess weight and T2DM, compared to a control group that received diabetes support and education (DSE) [357]. The ILI group used two MR in the first 20 weeks and one MR from week 21 on [357]. After one year, the ILI group achieved a mean loss of 8.6% of initial weight and 20.4% increase in physical activity compared to a mean loss of 0.5% of initial weight and a 5% increase in physical activity in the DSE group [358]. The superiority of the results in the intervention group was maintained after 4 [359] and 8 years [305].

More recently, the DIRECT and DIADEM-I trials evaluated the effect of a weight-loss program on T2DM remission in subjects with excess weight [360, 361]. In both studies, participants were randomized in two groups: a control group, that received standard diabetes care with less intensive follow-up, and an intervention group, that received total diet replacement for the first 12 weeks, followed by structured food reintroduction with a balanced diet for weight maintenance until the end of the study. In both studies, at 12 months, the intervention groups presented significantly greater weight loss (− 10 kg in the DIRECT trial and − 11.98 kg in the DIADEM-I trial) compared to the control groups (− 1 kg in the DIRECT trial and − 3.98 kg in the DIADEM-I trial), and a higher proportion of participants in the intervention groups achieved diabetes remission (46% vs. 4% in the DIRECT trial and 61% vs. 12% in the DIADEM-I trial) [360, 361].

A randomized study investigated the effect of two different weight-loss approaches in menopausal women with obesity class I and II [362]. Fifty women were submitted to moderate calorie restriction with a food-based diet, with energy intake restricted by 25–35% relative to requirements (control group) for 12 months, and 51 women were advised to follow a severe calorie restriction with a total meal replacement diet, with energy intake restricted by 65–75% relative to requirements (intervention group) for 4 months, followed by moderate calorie restriction for an additional 8 months. All participants were encouraged to engage in 30 to 60 min of daily moderate-to-vigorous physical activity per day, without supervision. At the end of 4 and 12 months, the intervention group lost more weight compared to the control group, but also presented greater loss of hip bone mineral density and more adverse effects related to the treatment (hemorrhoids, gallstones, and hair loss), results that were maintained after 36 months [363].

A sub-analysis of an international multicentric RCT evaluated the use of MR compared to lifestyle change on weight loss and food intake of subjects (n = 463) with BMI between 27 and 35 kg/m^2^ and metabolic risk factors [364]. All participants received printed guidance on healthy eating and were encouraged to increase their physical activity level. The intervention group (INT) was asked to replace one of the daily meals with a high-protein (53.3%) MR for 23 weeks, followed by lifestyle change for weight maintenance without the use of MR. After 12 weeks, the INT group (n = 82) had greater protein intake, lower fat and carbohydrate intake and greater weight loss than the control group (n = 37). However, with 52 weeks of follow-up, there was no statistical difference between the groups [364].

A systematic review and meta-analysis assessed the effect of MR vs. control on long-term weight loss (> 1 year) in individuals with excess weight [365]. A total of 23 RCTs were included, with 8253 participants (Intervention: n = 4411; Control: n = 3852) with average BMI of 34.5 kg/m^2^. The intervention groups were advised to use one or more MR per day, and some had additional support programs for weight loss. The control groups had minimal guidance, meal plans, or meal plans plus support for weight loss. In one year, weight loss was greater in the intervention groups. The results of the few studies that assessed weight loss at 2 or 4 years were also favorable for the intervention groups [366].

According to the Academy of Nutrition and Dietetics [163], MR can help to structure a LCD and increase diet adherence, by reducing problematic food choices and the challenge of making decisions. Besides that, they can also increase adherence through portion control, restriction of food variety and greater convenience [163]. On the other hand, MR do not promote mastication, a mechanism that can help to reduce food intake [365] through appetite reduction, by increasing cholecystokinin (CCK) levels and reducing ghrelin levels [365, 367]. In addition, evidence suggest that solid foods promote greater satiety than liquids [368, 369]. Moreover, it is important to highlight that, in the studies evaluating MR, these are often given to the participants at no cost, which could facilitate the adherence to this strategy.

Given that obesity is a chronic disease with lifelong treatment and that eating involves pleasure and social interactions, the individual must learn and feel capable to make better and varied food choices, as well to control the portions consumed throughout the treatment. Therefore, MR can be part of a healthy diet, especially for individuals that need the convenience and ease that they provide. However, this should be an individual recommendation based on the routine and habits of the patient, always taking in consideration the cost of MR, as well as its effect on satiety, that can vary from one person to another. When prescribing MR, it is essential to pay attention to the composition of the product, avoiding those with high amounts of sugars such as maltodextrin (except for modified maltodextrin, of low absorption), sucrose, and fructose, high amounts of saturated and/or trans fats, and low amount of fiber. Finally, it is important to emphasize that the benefit of the use of MR was shown in studies with up to one year of follow-up, with more studies being necessary to assess the long-term effect.

**Table:** 

Statement	Class of recommendation	Level of evidence
Meal Replacements can help to structure a LCD and increase diet adherence	IIa	A

## Low-carbohydrate diets

Obesity is a complex disease; therefore, treatment is not simple. Thus, a large number of approaches come up in order to increase the number of strategies to fight this disease. One of these approaches is the carbohydrate-restricted diet that became popular since the publication of the book Dr. Atkins' Diet Revolution, in which the intake of bread, pasta, grains, fruits and starchy vegetables is limited and the intake of animal protein, fats and butter is allowed without restriction. This type of diet has been spread and studied for many years [370].

Nevertheless, the results from RCTs are still divergent. Many studies have shown the superiority of low-carb diets to promote weight loss compared to low-fat diets, however these studies measure short-term weight loss, usually for less than one year [371–374]. It was also observed that low-carb diets contribute to increased TEE [375], adiponectin levels [374], fat oxidation [373], HDL-C levels [13, 376–380], to lower triglyceride levels [13, 371, 372, 381, 382] and to a greater reduction of abdominal circumference [379, 383]. On the other hand, other studies have described an increase in plasma LDL-C with this type of diet [373, 380].

Just as many RCTs have shown that low-carb diets can be more effective than low-fat diets for weight loss, several short-term (less than one year) [376, 378–380, 383, 384] or long-term (one year or more) [13, 377, 381, 382, 385] RCTs have shown that weight loss is similar with both diets. Besides that, it was demonstrated that low-fat diets are more effective to improve endothelial function [386] and ensure better satiety assessments throughout the day [387].

A meta-analysis assessed the effect of low-carb diets (very low in carbohydrates or moderately restricted) for weight loss in adults with excess weight, compared to a control diet with 46 to 55% of TEI from carbohydrates, 15 to 20% of energy from protein and 20 to 30% of energy from fat, or to the habitual intake [388]. Fourteen RCTs were included, with a total of 1805 participants (906 submitted to low-carb diets and 899 to control diets). The follow-up length varied from 2 to 24 months. In the studies lasting less than 12 months, weight loss and reduction of body fat were greater in patients in the low-carb group. In the studies lasting more than 12 months, there was no significant difference in weight loss between the groups, but still there was a greater reduction in body fat with carbohydrate-restricted diets [388].

A systematic review assessed 10 systematic reviews with meta-analysis and 2 systematic reviews without meta-analysis [370]. Only studies that compared any type of low-carbo diet to a control diet in individuals with excess weight were included, without concomitant use of medical therapy or physical activity. This review aimed to show differences between methods, study quality and the conclusion of published systematic reviews, as well as to investigate weight-related outcomes reported in the meta-analyses according to the quality of the study. It was observed that, in meta-analyses of studies with greater carbohydrate restriction and shorter duration, weight loss was significantly higher compared to hypocaloric or low-fat diets. However, when the studies had lower carbohydrate restriction and longer duration, the effect was attenuated. Regarding the quality of the meta-analyses, low-carb diets were considered superior for weight loss when the study quality was critically low, while results were inconsistent in meta-analyses of moderate quality and there was little or no difference in high-quality meta-analyses. Therefore, it can be concluded that energy intake lower than energy expenditure leads to weight loss, regardless of the macronutrient composition of the diets [370].

The divergent outcomes observed in systematic reviews can be a consequence of several factors. There is variation in the methods, contributing to low methodological quality. The definition of low-carb diet also varies, ranging from 20 to 120 g (6 to 45% of energy from carbohydrates), there are differences in inclusion and exclusion criteria, in the number of databases searched and in the assessment of risk of bias [1]. In addition, the studies not always measure the degree of diet adherence of the participants [389].

An important point mentioned by Churuangsuk et. al. [370] is that most of the meta-analyses did not report adverse effects [370]. In fact, few RCTs have reported these data, but the literature mentions adverse effects such as: obstipation, headaches, halitosis, muscle cramps, diarrhea, and weakness [390].

Low-carb diets promote weight loss in short and medium duration (3 to 6 months) and seems to be safe in the short term; weight loss might be related to the restriction of food choices, monotony and simplicity of the diet, and also to the greater satiating effect of protein. However, low-carb diets do not promote more weight loss compared to other diet types in long-term studies. Furthermore, longer studies with larger samples are needed to enable the assessment of energy balance, body composition, adverse effects, diabetes and cardiovascular risk factors, bone and kidney health, and to ensure nutritional adequacy during the weight maintenance phase [389], beyond the assessment of the effect of the low intake of whole grains, fruits and fiber inherent to low-carb diets in the risk of some types of cancer [370].

**Table:** 

Statement	Class of recommendation	Level of evidence
1. Low-carb diets promote weight loss in short and medium duration (3 to 6 months)	IIa	A
2. Low-carb diets do not promote more weight loss compared to other diet types in long-term studies	IIa	A

## Ketogenic diet

In the last years, the ketogenic diet has regained notoriety as a strategy for the treatment of obesity [391] and T2DM [392], possibly due to the spread of diets that promote fast short-term weight loss, such as low-carb, Paleolithic, and Atkins diets [392, 393]. In spite of the acute effect on weight loss observed in some studies, it is important to assess probable risks, possible benefits and its applicability, in order to reduce or avoid possible damages to patients [392].

The association between carbohydrate intake and obesity has gained strength in virtue of the recommendation for the US population to reduce fat intake because of the high prevalence of CVDs in the 1960–1970s [394]. Consequently, the decrease in fat consumption resulted in increased carbohydrate consumption [395], which coincided with the rise in obesity when the previous period was compared to 2007–2008 [396]. Despite the coincidence, this association should be interpreted with caution, since carbohydrate intake increased from 44 to 48.7% of TEI, while fat intake went from 36.6 to 33.7% of TEI [397]. These percentages do not characterize a high-carbohydrate or even a low-fat diet. Actually, what was observed was an increased intake of sugars, concomitant to an additional intake of approximately 300 kcal/day [395]. Therefore, carbohydrates cannot be blamed in isolation for the increase in obesity among the US population.

The percentage of fat used in studies that investigated the effect of ketogenic diet on the treatment of obesity ranges from 60 to 70% of total energy [393, 398]. As for carbohydrates, they provide 5–10% of total energy [397] or less than 50 g per day [398, 399]. Carbohydrate restriction in amounts less than 50 g/day induce the hepatic production of ketonic acids, such as acetoacetate and β-hydroxybutyrate, from the oxidation of fatty acids. These mediators will be used as an alternative energy source by extrahepatic tissues, replacing glucose [398].

Some authors justify the use of ketogenic diet for the treatment of obesity based on the carbohydrate-insulin model, that theorize that diets high in carbohydrates induce: (1) hyperinsulinemia and decreased adipose tissue lipolysis, thereby reducing fatty acid release [400]; (2) fat storage in the adipose tissue and decreased plasma glucose levels; (3) decreased oxidation of fatty acids in metabolically active tissues, such as heart, muscle, and liver; (4) increased calorie intake and obesity [400–403]. Therefore, the development of obesity would be the result of fat accumulation in the adipose tissue promoted by insulin [401], associated with a decrease in energy expenditure and an increase in calorie intake, in order to compensate for the lack of energy substrates from other tissues, or a supposed “starvation” condition, subverting the positive energy balance from cause to consequence [400, 403]. The increase in appetite and the reduction in energy expenditure would reflect a compensation pathway of the organism to counterbalance the sequestration of energy substrates by the adipose tissue, which become unavailable to meet the energy demands of other tissues [403]. In this context, some authors argue that the ketogenic diet reduces plasma insulin levels, limiting the storage of fat and glucose in the adipose tissue, leading to increased satiety, lean mass preservation, and increased energy expenditure, contributing to weight loss [400, 401, 403].

In spite of this theoretical description, the carbohydrate-insulin model has been largely refuted, because even though insulin plays an important role in the regulation of body fat, the model emphasizes only the direct action of insulin in the adipose tissue after the consumption of a high-carbohydrate diet [404]. However, it is known that the action of insulin in obesity must be comprehended considering its pleiotropic actions in multiple organs, mainly driven by factors that are independent of carbohydrate intake [404]. Thus, the mechanisms that explain the effects of insulin on adiposity are more complex than those proposed by the carbohydrate-insulin theory that is considered simplistic by some authors, since it neglects the imperative of excessive calorie intake and the rise in plasma insulin levels observed in obesity [405, 406].

Albeit its popularity, controlled studies in humans do not corroborate the alleged benefit attributed to the carbohydrate-insulin model [407, 408]. A trial conducted with adults submitted to a ketogenic diet compared to a diet with 50% carbohydrates for four weeks each showed that the loss of fat mass was diminished with the transition from the high-carbohydrate to the ketogenic diet [407]. The authors attribute this result to the increase in muscle protein utilization during the ketogenic diet, as demonstrated by the increased excretion of urinary nitrogen [407]. An elegant randomized controlled trial compared the effect of a 2-week period of a ketogenic diet (75.8% fat, 10.0% carbohydrate) or a plant-based diet (10.3% fat, 75.2% carbohydrate) on calorie intake and body composition of 20 subjects [408]. Both diets were minimally processed and participants were instructed to eat ad libitum. There was no difference in food intake, hunger and satiety between the diets, but the ketogenic diet led to higher energy intake. Besides that, ketogenic diet resulted in greater weight loss, however due to the loss of lean body mass (assessed by DXA). At the end of each period the participants were submitted to an oral glucose tolerance test (75 g) and the authors observed that insulin sensitivity was lower after the ketogenic diet compared to the low-fat diet [408]. It is worth mentioning that the findings of this study were inconsistent with the carbohydrate-insulin model [409]. Moreover, several authors have not confirmed the benefits of high-fat diets on food intake, because they do not contribute to increase satiation and satiety [408, 410, 411].

A meta-analysis assessed the efficacy of a ketogenic diet in metabolic control of patients with overweight or obesity, with or without T2DM [22]. Thirteen RCTs were included, and the authors concluded that the ketogenic diet led to greater weight loss compared to a low-fat diet in short-term studies (3–6 months). However, the difference in weight loss was not sustained in long-term studies (24 months). It is worth to highlight that weight deficit was of less than one additional kilogram relative to the other groups studies, a result that, although statistically significant, is not clinically relevant [392]. With regard to blood lipids, ketogenic diet led to increased LDL-C, lower plasma triglycerides and increased HDL-C [412]. Despite being established in the literature that a diet high in saturated fat raises HDL-C, it is known that this increase is not sufficient to outweigh the detrimental effect promoted by the increase in LDL-C, lipoproteins that promote atherogenesis. Furthermore, it is known that the functionality of HDL can be compromised by a high-fat diet, especially when rich in saturated fatty acids, because of the increase in inflammatory compounds in this particle [413].

A systematic review and meta-analysis discussed the data from 12 studies, involving 801 healthy adults, comparing the effect of different dietary strategies: (1) very-low-calorie ketogenic diet (VLCKD, < 800 kcal/day), (2) ketogenic diet very low in carbohydrates, (3) very-low-calorie diet (< 800 kcal/day), (4) low-calorie diet, and (5) isocaloric ketogenic diet [414]. Average weight loss with the VLCKD was 10 kg in the studies up to 4 weeks and 15 kg in studies lasting 4 weeks or more. Weight loss during the ketogenic phase remained stable in the subsequent follow-up until two years. A reduction in waist circumference was observed as a function of weight loss. The prevalence of patients that interrupted this diet (7.5%) was similar to that observed with the hypocaloric diet. Although the authors concluded that the use of VLCKD can be a strategy for the management of excess weight and biochemical and cardiovascular parameters, they emphasized the concern with regard to the safety of following this diet, since it can be harmful for people with hydro electrolytic disturbances, type 1 diabetes mellitus (T1DM), and cardiovascular disease. It is noteworthy that the study showed no difference in weight loss when VLCKD were compared to very-low-calorie diets [414].

Lukkonen et al. [415] assessed the impact of a ketogenic diet on non-alcoholic fatty liver disease (NAFLD) and observed that, in spite of metabolic improvements, there were adverse effects only 6 days after the beginning of the diet. The AST/ALT ratio increased by approximately 34% during the diet, suggesting that very fast weight loss can induce a transient hepatocellular injury. In addition, metabolic alterations induced by the ketogenic diet are similar to those observed during fasting. In this group of patients there was a 13% increase in protein oxidation, even without altering protein intake, demonstrating an increased protein catabolism during this dietary intervention [415].

A systematic review and meta-analysis that evaluated studies with resistance training associated with a ketogenic diet also showed negative effects on lean body mass, even with an additional period of resistance training [416]. Thus, weight loss induced by the ketogenic diet can result in decreased muscle mass, reiterating the lack of advantage of following this diet in comparison to others with higher amounts of carbohydrates [417].

It is important to emphasize that the undesirable effects of ketogenic diets include: low intake of fiber and whole grains, dehydration, hypoglycemia, lethargy, halitosis, nausea, vomiting, hair loss, among others [418]. Also, ketogenic diets increase LDL-C, potentially increasing long-term cardiovascular risk [418]. Another important adverse effect is the occurrence of ketoacidosis in individuals T1DM, that might develop even after few days after the beginning of this diet [419].

Ketogenic diet is associated with negative effects on gut microbiota, since it results in reduced microbial diversity due to the low intake of carbohydrate sources rich in fiber, with less production of short-chain fatty acids [420]. These fatty acids improve insulin sensitivity in the adipose tissue and contribute to increased satiety [420].

Due to the need of a long-term treatment for obesity, physicians and dietitians must carefully assess the risks of following the ketogenic diet according to scientific evidence [392]. The ketogenic diet is associated with low consumption of water-soluble vitamins and low plasma calcium levels [421]. The motivation for the indication of this dietary practice exceeds the current evidence that would support its recommendation and the potential risks of the chronic follow-up of the ketogenic diet are well established [392].

**Table:** 

Statement	Class of recommendation	Level of evidence
The ketogenic diet should not be recommended for the treatment of obesity as it does not promote a balanced diet or favor adherence to healthy eating habits	III	A

## Low-glycemic index diet

The concept of glycemic index (GI) was introduced in 1981 with the aim of classifying foods according to their effect on post-prandial glycemia [422].

GI is a physiological measure of the impact that the type of dietary carbohydrate has on the elevation of blood glucose [291, 423]. It is defined as the glycemic response curve after the consumption of a test food in relation to the glycemic response observed after the ingestion of a standard food (glucose or bread) [401]. The GI of a specific food or meal is primarily determined by the nature of the carbohydrate consumed and by other factors that affect food digestibility and insulin secretion [401].

The GI classifies carbohydrate food sources according to the magnitude of the elevation of blood glucose induced in comparison with a standard amount (50 g) of a reference food (pure glucose or white bread) [424]. Thereby, some authors classify the GI of foods as low (≤ 55), medium (56–69), or high (≥ 70) [424, 425]. The glycemic load (GL), in turn, is calculated by multiplying the GI by the available carbohydrate (g) per serving, and then dividing by 100 [424, 426].

An important systematic review entitled “The fourth edition of the International Tables of Glycemic Index and Glycemic Load Values Lists”, published in 2021 [427], evaluated data from 253 RCTs and cohort studies and listed the GI of over 4000 foods, showing that foods with low-GI (≤ 55), such as dairy products, legumes, fruits, and pasta, had consistent values in the studies. However, there was variability in the response to foods associated with a medium or high-GI [427].

An important point to reflect on is the clinical applicability of the use of tables of GI, since most of the time foods are not eaten in isolation, they can be eaten *in natura* or in preparations, together with other food sources. Besides that, whether in isolation or as part of a preparation, they can comprise a meal. Therefore, the addition of other nutrients such as fats and proteins can significantly affect the GI in response to carbohydrate consumption. In this context, the GI of baked potatoes is 78, while the GI of French fries is 63 [428]. The GI of a regular croissant is 67, while for a croissant prepared with a lot of fat, the GI is 45; a pancake prepared with refined flour and 1 g of oil has a GI of 57, while the one made with whole wheat flour, cheese and butter has a GI of 37 [427]. Thus, the fact of presenting a lower GI does not mean that the food or preparation is healthier from a cardiovascular point of view, and can even contribute to an increased calorie intake [427].

Another relevant issue is the variability in GI response observed for the same foods grown in soils with different compositions [427]. Finally, there is also variation in the glycemic response depending on individual characteristics, such as body weight or even the degree of insulin sensitivity, which can vary in the same individual throughout the day. In this context, it is important to emphasize that the meta-analysis that lists the GI of foods excludes studies conducted in individuals with glucose intolerance or with Diabetes Mellitus [427].

The 2021 guidelines from the American Diabetes Association mention that the literature concerning the use of GI in diabetes management is complex and controversial, with varying definitions of low, medium or high GI [291]. They also argue that studies assessing the use of GI in diabetes management have presented conflicting conclusions regarding the effect of GI both on fasting glucose levels and on glycated hemoglobin [291]. A systematic review of 73 RCTs showed no impact of the GI on glycated hemoglobin [429]. In contrast, another systematic review of 54 studies including children and adults with T1DM and T2DM revealed that the use of GI led to a 0.15% reduction in glycated hemoglobin, with no change in fasting insulin, and did not influence insulin needs [430]. A previous meta-analysis study that used data from the Cochrane Library showed a 0.5% reduction in glycated hemoglobin when comparing the consumption of low-GI with high-GI foods. In this review, 11 studies were included, with a total of 402 adult individuals with T1DM and T2DM [431].

With regard to the impact of GI on body weight, an extensive review including randomized controlled trials and observational studies was conducted [429]. Part of the review assessed nine cross-sectional conducted in different populations, with or without diabetes, with BMI and waist circumference as the primary outcomes, and showed no association between the consumption of foods with different GI levels with these parameters. Among the studies, an investigation conducted in a Japanese population (n = 3931 adults) showed that lower GI was associated with lower BMI (− 0.7 kg/m^2^), comparing the lowest with the highest quintile [429, 432]. In another study, conducted with 8195 adults (BMI = 18.5–60 kg/m^2^), the difference in BMI between the lowest and the highest GI tertile was from 0.7 to 1.0 kg/m^2^, but only in women [433]. Data from cross-sectional studies were inconsistent in terms of the direction and the intensity of the association between GI and body weight [429].

Concerning waist circumference, in studies conducted in large populations of healthy individuals the GI did not influence this measure [429, 434, 435]. However, smaller studies with individuals with T2DM (n = 175 and n = 238) reported a positive association between waist circumference with GI [436] and GL [437].

Another arm of this meta-analysis evaluated eight intervention studies with a duration of 8 weeks to 18 months. The results are insufficient to support the benefit of indicating low-GI diets for weight loss. Although short-term studies have suggested a benefit of consuming low GI-foods on weight loss [438, 439], highly controlled studies conducted over a longer period have suggested that the manipulation of the GI did not interfere with weight loss [440–443].

The OmniCarb Randomized Clinical Trial was a controlled crossover study [444] conducted in 166 individuals with overweight randomized to 4 DASH-style diets varying by GI: 1. High-GI (65% in the glucose scale), rich in carbohydrates (58% of daily calories); 2. Low-GI (40%), rich in carbohydrates; 3. High-GI, low in carbohydrates (40% of daily calories); and 4. Low-GI, low in carbohydrates. Each diet was consumed over a 5-week period, and all participants who completed at least 2 diet periods were included. All meals and snacks were offered to the participants. Calories were adjusted to maintain initial body weight and, at the end of the trial, participants lost an average of 1 kg, without difference between each diet type. The different diets did not result in improvements in any of the primary outcomes evaluated, including those associated with cardiovascular risk, such as LDL-c, HDL-c and blood pressure, as well as in insulin resistance. A modest reduction in plasma triglyceride levels was observed (from 91 to 86 mg/dL, − 5%, p = 0.02) [444].

An important review published in 2021, entitled “Does Glycemic Index Matter for Weight Loss and Obesity Prevention? Examination of the Evidence on “Fast” Compared with Slow Carbs” [445] used data from the Cochrane database of observational studies that reported association between BMI and GI and meta-analyses of randomized controlled trials that compared the effect of low and high-GI on weight loss. A total of 43 cohorts from 34 publications with 1,940,968 individuals were used in the analysis and the data revealed no consistent difference in BMI when comparing high versus low-GI. Of 27 studies, 70% showed that BMI was not different when comparing high versus low-GI. The results of 30 randomized controlled studies showed that low-GI diets were not better for the reduction in weight or body fat [445]. Among the studies evaluated, only one meta-analysis of 10 randomized controlled studies, with 2344 participants, compared the effect of low versus high-GI diets on waist circumference, and showed no difference between the diets [446].

The International Scientific Consensus Summit from the International Carbohydrate Quality Consortium showed that it is possible that the GI can contribute to weight management, but more studies are necessary to confirm this [447]. In this document, the authors also concluded that the results of studies with children and adolescents suggest a very modest role of GI in the prevention and treatment of obesity in this age group [447].

Based on the results of observational cohort studies and meta-analyses of randomized controlled trials, the evidence is scarce to affirm that a low-GI food/meal is superior to a high-GI one for weight loss [445].

**Table:** 

Statement	Class of recommendation	Level of evidence
Glycemic Index, as a measure of carbohydrate quality, appears to be a minor determinant for body weight, weight loss and obesity prevention	IIb	B

## Influence of meal frequency and timing on obesity

In the last years, diets with intermittent caloric restriction (intermittent fasting) have been pointed as a strategy to promote weight loss and amelioration of the metabolic profile and have gained popularity and attention in the media and among patients. Intermittent fasting (IF) is the term used for plans that cycle between periods of free food intake and periods of fasting (16–24 h) or severe caloric restriction (intake of 25% of the energy needs, usually in a single meal during the feeding window), or plans that allow a 4 to 8-h feeding window with ad libitum energy intake followed by fasting during the remaining hours [448, 449].

It is worth mentioning that IF does not require changes in eating habits or the adoption of a healthy dietary pattern, restricting only the frequency and/or time of calorie intake. Besides that, most of the studies that assessed the impact of IF on weight loss had no control group, included a small number of participants and were of short duration (< 3 months) [450, 451].

Bhutani et al. [452] showed that, in individuals with obesity, alternate day fasting (ADF) led to greater weight loss compared to the control group; however, individuals in the control group were advised to maintain their habitual diet, without calorie restriction. In individuals with normal weight and overweight (BMI 20–29 kg/m^2^), ADF resulted in greater loss of weight and fat mass compared to the control group. Yet again, individuals in the control group maintained their habitual diet with higher energy intake (around 700 kcal/day) compared to the ADF group [453].

In women with overweight or obesity with a family history of breast cancer, but without previous disease, IF reduced percentage body fat, fasting insulin, and HOMA compared to continuous calorie restriction. It is worth pointing out that this difference was not seen at study month 4, and that weight loss and improvement in metabolic parameters were similar between groups. Regarding the adequacy of the intake of specific nutrients, fiber, zinc, magnesium and selenium deficiencies were more apparent in the IF group compared to continuous calorie restriction [454].

In individuals with obesity, ADF was not superior to calorie restriction with regard to weight loss and maintenance in a study with 12 months follow-up. However, dropout was higher in the ADF group compared to the other groups, which might suggest greater difficulty to follow this type of diet [455]. Similarly, in individuals with obesity and insulin resistance, ADF reduced fasting insulin and HOMA-IR at 6 and 12 months compared to continuous calorie restriction, regardless of weight loss, which was similar for both groups [456].

In a recent systematic review of randomized trials lasting from 12 weeks to 12 months that investigated the effect of IF on weight loss, body composition, and glycemic control of individuals with obesity and T2DM, the results showed that IF was not superior to continuous calorie restriction regarding the study paremeters [457].

The TREAT study explored the effect of 16:8-h time-restricted eating (abstaining from food intake for 16 h followed by ad libitum intake for 8 h) on weight loss and metabolic risk markers in individuals with overweight or obesity, compared to a diet with three structured meals per day. None of the participants received recommendations concerning calorie intake, macronutrient intake or physical activity. The 12-week study showed that the modest decrease in weight was similar between groups and there was no difference in the markers of metabolic risk [458].

Because weight loss per se is responsible for significant metabolic improvements, a relevant study assessed the impact of a 6-h feeding window on markers of cardiometabolic risk, regardless of weight loss. To this end, eight individuals with overweight and pre-diabetes had their food and calorie intake monitored, paired, and divided in three meals to be consumed in a 12-h feeding window (control) or in a 6-h window (early time restricted feeding—eTRF), with the last meal consumed until 3:00 p.m. The results showed that eTRF improved insulin sensitivity, β cell responsiveness and blood pressure compared to the control group, independent of weight loss. On the other hand, in the eTRF group the reduction in cholesterol levels was lower and there was an increase in fasting triglyceride levels, possibly due to the longer fasting duration. In addition, participants reported that the main difficulty was the challenge of eating within 6 h each day [459]. It is worth mentioning that from 130 individuals screened for eligibility, 15 met all the eligibility requirements and only 8 completed the protocol, which predicted only 5 weeks in each study arm.

Therefore, IF presents similar weight loss results to meal plans with daily calorie restriction, since it also promotes an energy deficit, and not because of the fasting period per se. However, long-term studies demonstrated lower adherence in comparison to continuous calorie restriction [460]. More controlled and long-term studies are needed to safely establish the effect of IF on inflammatory and cardiometabolic parameters.

In the last years, studies have been showing the importance of the circadian rhythm to the maintenance of cardiometabolic health. Circadian rhythm operates as a biological clock, conferring adaptative advantage for living organisms in the 24-h period determined by the Earth’s rotation. Several physiological processes that exhibit daily variation are controlled by the circadian rhythm, including glucose, lipid and protein metabolism, as well as hormone secretion and cardiac function [461, 462]. Thus, alterations in the sleep/wake pattern or in the eating routine (fasting/postprandial) can disrupt the circadian rhythm and impair the functioning of the organism and overall health, increasing the risk of obesity, insulin resistance, and cardiovascular disease [463, 464].

In this sense, McHill et al. [465] investigated the food intake and sleeping pattern of 110 patients and observed that those who consumed most of their calories closer to melatonin onset, which heralds the beginning of the biological night, presented greater accumulation of body fat, independent of diet composition. A study with 1245 individuals with 6 years of follow-up showed that individuals who consumed ≥ 48% of daily calories at dinner had increased risk of obesity, metabolic syndrome and fatty liver disease, independent of total calorie intake and macronutrient distribution [466]. In another study, it was demonstrated that individuals who ate lunch late (after 3:00 p.m.) had lower and smaller weight loss during the treatment [467]. A systematic review on the subject showed that the consumption of a higher percentage of calories in late meals can negatively affect body weight and insulin action [468].

With regard to meal frequency and body weight, the Adventist Health Study 2 analyzed data from 50,660 participants and showed a positive correlation between the number of daily meals and the relative increase in BMI; in contrast, there was an inverse association in individuals who had a long overnight fast. Furthermore, consuming breakfast and eating the largest meal in the morning were considered effective habits for preventing long-term weight gain [469]. Data from the Health Professional Follow-up Study showed an inverse correlation between breakfast consumption and the risk of 5-kg weight gain during 10 years of follow-up. Yet, an increasing number of eating occasions in addition to three standard meals was associated with a higher risk of weight gain [470]. This is possibly related to the consumption of snacks of low nutritional quality [471]. However, snacking can occur in the context of healthy food choices, and therefore not be associated with weight gain [472].

Consequently, it becomes evident that regardless of the number of meals, it is important to promote a healthy eating habit, tailoring the TEI to the individual’s needs, whether for weight loss or maintenance.

Obesity is a chronic disease that requires lifetime support and treatment. Thus, it is essential that the proposed meal plan is compatible with each patient’s lifestyle. Meal plans that complicate the routine and social interactions or that aren’t matched to the individual’s lifestyle tend to be successful for a short time, favoring weight regain. In addition, the approach of the professional must support lifestyle change, encourage healthy eating habits and the practice of physical activity, as well as develop strategies to facilitate diet adherence, in order to promote sustainable long-term weight loss.

**Table:** 

Statement	Class of recommendation	Level of evidence
1. Intermittent calorie restriction presents similar weight loss results as meal plans with continuous calorie restriction	IIb	A
2. Weight loss is induced by the energy deficit, and not by the fasting period per se or the number of daily meals	IIb	A
3. Late consumption of most of the daily calories can negatively impact body weight	IIb	B

## Phytotherapics

According to the resolution RDC 26/2014, phytotherapics medicines are those obtained with the exclusive use of active vegetable ingredients which safety and efficacy are based on clinical evidence and are characterized by the consistency of their quality [473]. In Brazil, phytotherapics medicines can be prescribed by dietitians with a degree of specialist in Phytotherapy [474]. Even though the dietitian is able to prescribe phytotherapics, this Position Statement reaffirms that there is no evidence of benefits of their use for the treatment of obesity.

Compounds such as *Ephedra sinica* [475–477] were not efficient to promote weight loss and induced psychiatric, gastrointestinal and cardiac adverse effects, including stroke [478]. *E. sinica* was evaluated in five studies (n = 546), lasting from 8 to 26 weeks, and the results showed statistically significant weight loss (− 0.58 kg, compared to placebo, however without clinical relevance [479]. Few experimental studies have shown that hydroxycitric acid (HCA), obtained from *G. cambogia* [480] inhibits lipogenesis [481], can suppress appetite and induce satiety [482] and reduce the sensation of hunger [483], although the possible effects on weight loss are not supported by human studies for the treatment of obesity [479, 480, 484].

With regard to the supplementation of fibers such as Psyllium (*Plantago *ovata) [485–487], guar gum (*Cyamopsis tetragonolobus*) [488] and glucomannan (derived from *Amorphophallus konjac* C. Koch) [489, 490], studies have shown no benefit in reducing body weight. Additionally, some individuals may present adverse events, predominantly related to the gastrointestinal system [488]. Chitosan appears to decrease the absorption of fat in animals [491–493], but this effect is not supported by human studies assessing weight loss. Besides, some of the studies included have serious methodological limitations [478, 494].

Other compounds, such as *Irvingia gabonensis* or African mango [495, 496], *Cordia ecalyculata* [497, 498], *Citrus aurantium* (bitter orange) [499–503], *Phaseolus vulgaris* (Phaseolamin or white beans) [479, 504], *Camellia sinensis* or green tea [479, 505, 506] and Yohimbine (*Pausinystalia yohimbe*) [478] also do not contribute to weight loss.

One of the limitations observed about phytotherapics is the presence of heavy metals above safe levels. One study assessed the level of metals in plant extracts prescribed as weight-loss supplements, among them *Camellia sinensis*, *Cordia ecalyculata*, and others. The metals more frequently detected were manganese, aluminum, and iron, with the highest concentration in *Camellia* sinensis [507].

Many studies on phytotherapy and weight loss have low methodological quality and the best quality ones do not show the effectiveness of these substances for weight loss.

**Table:** 

Statement	Class of recommendation	Level of evidence
There is no recommendation to use phytotherapics for weight loss	III	B

## Caffeine

Caffeine is an alkaloid in the methylxanthine family and is usually associated with theophylline and theobromine. It has stimulating and psychoactive properties and is naturally found in more than 60 species of plants. The most common dietary sources are coffee, some teas, cocoa-based products and cola-based soft drinks [508].

The average intake of caffeine of Brazilian and American adults is 160 mg and 180 mg, respectively, with coffee as the main source [509, 510]. In the adult population, caffeine intake of up to 400 mg/day is not associated with adverse effects (cardiovascular, cancer incidence, bone density and behavioral changes). Evidence suggests that a daily caffeine intake of up to 300 mg by healthy pregnant women does not cause harmful effects [510, 511], while caffeine intake is discouraged for children and adolescents [510, 511].

Since it was isolated, caffeine has been added to soft drinks, energy drinks, supplements and drugs, becoming the most widely used psychoactive substance in the world [510]. Caffeine acts as an antagonist of the adenosine receptor, reverting the inhibitory effect of adenosine and resulting in increased central release of noradrenalin, dopamine and serotonin, as well as increased catecholamines and plasma renin activity [512]. Furthermore, caffeine has a bronchodilator effect and increases blood pressure and renal excretion of water and sodium [512].

In rats, chronic caffeine intake attenuated weight gain and the increase in visceral fat and insulin resistance induced by a high-fat diet. Besides that, prolonged caffeine intake reduced the serum levels of catecholamines, suggesting a decrease in sympathetic tone, an opposite effect to that observed with acute caffeine treatment [513].

In humans, a single dose of 250 mg of caffeine increased the levels of adrenalin and noradrenalin, plasma renin activity and blood pressure 1 h after the ingestion [514], which might be of clinical relevance for habitual consumers, especially those with low caffeine tolerance [515].

Caffeine intake combined with ephedrine, but not alone, increased brown adipose tissue thermogenesis in animals [516]. In humans, caffeine potentializes the thermogenic effect of ephedrine, a sympathomimetic agent that stimulates adrenalin release. Acute ephedrine intake combined with caffeine (20 mg/200 mg) induced a modest increase in energy expenditure (around 30 kcal in 3 h), while increased systolic and diastolic blood pressure [517].

The intake of coffee or caffeine does not seem to influence dietary energy intake and macronutrient distribution [518]. A study on healthy adults assessed the effect of increasing doses of caffeine (0; 0.1 or 3 mg/kg) on ad libitum breakfast and found no difference in appetite and food intake, regardless of the BMI [519]. Similar results were reported in a study that showed that an intake of two equal doses of caffeine totalizing 4 mg per kg per day, in healthy subjects, did not influence energy intake, appetite, satiety, and gastric fulness sensation [520].

A randomized double-blind crossover study studied the effect of a daily intake of 103 mg of caffeine over 14 days on the energy metabolism of 12 healthy men [521]. Caffeine intake until noon did not alter sleep architecture. Also, there was no difference in 23-h energy expenditure measured by indirect calorimetry. However, the respiratory quotient was lower with caffeine intake compared to placebo, suggesting an increased fatty acid oxidation and decreased carbohydrate oxidation, without any difference in protein catabolism (assessed by urinary excretion of nitrogen) over the 23-h period [521].

Several studies reported adverse effects of excessive caffeine intake, specially from the consumption of energy drinks, supplements or caffeine-containing drugs. In the United States, more than 13,000 cases of adverse effects related to the consumption of caffeinated energy drinks were reported between 2008 and 2015 [522]. The most common symptoms were tachycardia, irritability, nausea, vomiting, psychiatric and nervous system alterations, and severe adverse effects [522]. Among the caffeine-containing products, those marketed as weight-loss products or “energetic” were associated with increased risk of severe adverse effects [523].

In Brazil, a study assessed the composition of food supplements marketed on the internet with the following claims: weight loss, fat burn, appetite reduction and metabolism booster. The analysis showed that part of the products had higher levels of caffeine than informed in the label. Besides that, many samples contained other stimulants and active substances that were not declared in the label, such as ephedrine, synephrine [524], clobenzorex, phenpromethamine, sibutramine, dipyrone, fluoxetine, amphetamine, phenproporex, and others [525].

In conclusion, the use of caffeine supplements should not be considered as a strategy for weight loss and obesity treatment, since caffeine seems to present an only modest effect on thermogenesis and does not influence appetite regulation and food intake. In addition, it has been demonstrated that caffeine-containing weight-loss supplements or “energetic” supplements can raise blood pressure and increase the risk of severe adverse effects.

**Table:** 

Statement	Class of recommendation	Level of evidence
The use of caffeine supplements should not be considered as a strategy for weight loss and obesity treatment	III	B

## Whey protein supplements

There are different types of whey protein, including whey protein concentrate (80% protein), whey protein isolate (100% protein), hydrolyzed whey protein (100% of partially hydrolyzed proteins), and native whey protein. It is present in several formulations including milk, milk powder, and specialized formulas enhanced with some amino acids, such as glutamine and/or branched-chain amino acids (BCAAs) [526]. Meta-analyses have shown that the supplementation of whey protein isolate led to reduction in weight, lean and fat body mass, and waist circumference in individuals with obesity or metabolic syndrome. Also, there was a beneficial effect on several indicators of glycemic control [527–529] and blood lipids, including triglycerides [527–529] and HDL-C [527–529], and on blood pressure [528, 529], thus improving cardiovascular risk factors. However, the difference in weight loss is not clinically relevant [527]. In the meantime, the subject remains controversial, since a previous meta-analysis did not find difference in weight or blood lipids with an increased protein intake [530].

Regarding the preservation of lean mass during diet-induced weight loss, whey protein supplementation does not seem to have a clinically significant effect [531, 532]. Nevertheless, positive metabolic effects are observed with whey protein supplementation, including an increased release of hormones such as GLP-1, leptin and cholecystokinin, and a decrease in ghrelin, alterations related to satiety, which can result in weight loss. In young women with obesity, the daily intake of 45 g of whey protein isolate, compared to 43 g of maltodextrin, reduced appetite and stimulated anorexigenic gastrointestinal peptides (PYY and GLP-1) after 2 hours [533]. The biological benefits of whey protein can also be associated with its nutritional compounds, particularly cysteine and BCAAs [526].

Whey protein can be recommended when there is a need to supplement protein intake, however, at the moment, results on the subject are controversial, not justifying its use in the treatment of obesity.

**Table:** 

Statement	Class of recommendation	Level of evidence
The prescription of whey protein is not indicated as a therapy for weight loss	III	A

## Coconut fat

Coconut fat, extracted from the flesh of mature coconuts, is presented in refined, bleached and deodorized oil or cold-pressed virgin or extra-virgin oil, a form of extraction that preserves the polyphenols present in coconut meat. Coconut fat cannot be classified as oil, due to its high content of saturated fatty acids [195].

The main producers of coconut fat are the Philippines, Indonesia and India [534], regions where coconut and its derivatives are an important source of income and calories for the local population [535]. Its use is widespread in the cosmetics industry, due to its emulsifying, bactericidal, and healing properties [536]. However, in recent years, the consumption of coconut fat has become quite popular in the media, and recommendations for its use have been claimed without well-conducted and conclusive studies presenting results that support their indications.

Among all fats (vegetable and animal), coconut has the highest percentage of saturated fatty acids (92%), with lauric acid being the one with the highest concentration (50%), followed by myristic (16%), palmitic (8%) and, in small amounts, caprylic, capric and stearic [537, 538]. In addition, coconut fat presents low concentrations of linoleic acid (18:2-ω-6) and does not contain linolenic acid (18:3, ω-3), both essential fatty acids [537, 538].

Saturated fatty acids are naturally present in the diet and have important biological functions; they act in signaling pathways, form cell membranes and are able to influence gene transcription and the stability of cell membrane proteins [539]. However, excessive consumption of saturated fat can have deleterious effects from a cardiometabolic point of view. It has already been shown that saturated fatty acids are capable of increasing plasma concentrations of LDL-cholesterol [540, 541] and increasing the presence of inflammatory components in HDL-cholesterol particles, compromising their functionality [413]. Moreover, other studies have corroborated the hypercholesterolemic action of lauric acid, both compared to safflower oil [542] and OO [543].

Therefore, it is recommended to adjust the consumption of saturated fat to less than 10% of TEI, with an additional reduction (less than 7% of TEI) for individuals with increased cardiovascular risk, replacing calories from saturated fat with unsaturated fat or complex carbohydrates [544].

Another promise attributed by the media to coconut fat is its use as a strategy for weight loss and increased satiety. It is possible that such statements are erroneously supported by the actions of medium-chain triglycerides (MCT), formed mainly by caproic (6:0), caprylic (8:0) and capric (10:0) acids, which are, almost in their totality, absorbed via the portal system linked to albumin and, as they do not require transport via chylomicrons, they do not increase triglyceridemia [545, 546]. It should be clear that MCT differs enormously from coconut fat, which contains more than 60% of its composition in the form of lauric and myristic acids (12:0 and 14:0). Unlike MCTs, lauric acid is mainly absorbed (70–75%) via chylomicrons [547, 548], especially when found in large concentrations.

A study conducted with 15 women with overweight tested the effect of the consumption of a meal with 25 mL of extra-virgin coconut oil (EVCO) or OO on energy expenditure, lipid oxidation, satiety and markers of cardiometabolic risk. EVCO consumption induced less fullness, total satiety and hunger suppression, compared to OO. There was no difference between EVCO and OO in relation to energy expenditure, lipid oxidation and postprandial effect on the metabolic parameters [549]. In addition, the consumption of meals enriched with coconut oil did not increase thermogenesis or satiety when compared to corn oil [550] or soybean oil [551].

An experimental study in Wistar rats compared the effect of EVCO, copra (refined coconut oil), olive, and sunflower oil on lipid metabolism. There was no difference in diet consumption or weight gain of the animals. However, compared to the other oils, consumption of EVCO reduced plasma and hepatic concentrations of total cholesterol, triglycerides and phospholipids, as well as the activity of hepatic lipogenic enzymes, and increased the activity of enzymes linked to lipid oxidation (assessed in cardiac tissue). It is worth mentioning that EVCO and copra had the same fatty acid profile, varying only in the concentration of polyphenols [552].

In C57/BL6 mice, the consumption of coconut oil induced less weight gain compared to the consumption of soybean oil [553]. However, the lipid profile of the animals, as well as the development of atherosclerotic lesion and inflammatory markers were not studied. These are important parameters to evaluate the safety of coconut oil, since weight loss in animals was also observed in a study with the use of trans fatty acids that promoted severe development of atherosclerosis, inflammatory profile, and diabetes [554]. Therefore, results from animal studies should be interpreted with caution.

It should also be noted that, among saturated fatty acids, lauric acid has a high inflammatory potential [555], due to its action on signaling pathways involving TLRs, which function is to recognize molecular patterns associated with pathogens, such as LPS, and alert the immune system, through a signaling cascade that culminates in activation of NF-kB and secretion of pro-inflammatory cytokines such as TNF-α, IL-1b, and IL-6 [556]. LPS contains lipids in its structure that interact with TLRs, being lauric (C12:0) > myristic (C14:0) > palmitic (C16:0), the main fatty acids in this fraction. Known as Lipid A, this structure is responsible for the endotoxic action of LPS [557]. The inflammatory action of saturated fatty acids, added to their hypercholesterolemic action, increases their atherogenic potential [554].

Coconut fat has a high percentage of saturated fatty acids, especially lauric acid. Its consumption is not related to weight loss and when consumed in excess, it increases the cardiometabolic risk.

**Table:** 

Statement	Class of recommendation	Level of evidence
There is no scientific evidence to support the indication of coconut oil as a weight loss strategy	III	B

## Probiotics

The consumption of probiotics for health promotion has largely increased over the last years. However, despite its popular use, the results from studies that assessed the efficiency of different strains and formulations are conflicting. Also, one of the key issues is the great variability both in the different formulations of probiotics and in the composition of the human microbiome, and the interaction between these factors [558, 559]. The well-known probiotic strains are represented by lactic acid bacteria of the genera Lactobacillus and Biffidubacterium [560]. Results from studies with probiotics are conflicting. L. *paracasei* F19 supplementation in mice reduced the size of adipocytes, white adipose tissue and serum leptin [561]. In humans, this strain did not demonstrate any metabolic effect in both postmenopausal women with obesity and school-age children [562, 563]. Supplementation with L. *rhamnosus* PL60 in mice led to a reduction in weight, fat mass, white adipose tissue and steatosis, without a decrease in food consumption [564]. In addition, insulin sensitivity was improved, probably due to the increase in adiponectin [565]. L. *casei* supplementation in mice led to a reduction in weight, BMI, lean mass, leptin, and blood glucose [566]. On the other hand, supplementation with fermented milk containing 108 CFU/mL, 65 ml 3 times a day for 12 weeks, was not able to lead to a reduction in BMI in individuals with metabolic syndrome [567]. A similar study, also conducted in individuals with metabolic syndrome receiving the same amounts of fermented milk, was also unable to demonstrate changes in BMI [568]. There was also no change in insulin sensitivity, low-grade inflammation and endothelial dysfunction, in addition to an increase in C-reactive protein (CRP) after 3 months of probiotic consumption [568].

*Bifidobacterium longum* supplementation in mice improved the immune system and the metabolic changes induced by endotoxemia and intestinal inflammation [569]. The use of *Bifidobacterium adolescentis* was also able to reduce visceral fat and hepatic steatosis in mice [570].

Positive effects were observed after the supplementation of *Bifidobacterium pseudocatenulatum* CECT7765 in mice for 7 weeks, such as reduction of the adipose tissue and improvements in insulin resistance, hepatic steatosis, and plasma levels of insulin, leptin, and IL-6 [571]. Another study that compared four different strains of Bifidobacteria: L66-5, L75-4, M13-4 and FS31-12 in rats, concluded that the effect can be very different according to the strain. For instance, the strain Bifidobacteria M13-4 induced weight gain, while B. L66-5 induced weight loss and the other two strains did not influence weight [572]. In turn, supplementation with *Bifidobacterium lactis* for a period of 45 days led to a reduction in body weight in individuals with metabolic syndrome [573].

In animal models, multi-probiotic supplementation was able to reduce Firmicutes and increase Bacteriodetes and Bifidobacteria, in addition to reducing weight, insulin resistance and central adiposity, improving the immune system, glucose tolerance and increasing production of GLP-1 and butyrate [574]. The mixture of *L.salivarius* 33, *L.rhamnosus* LMG S-28148, and *B.animalis subsp. lactis* LMG P-28149 was tested in mice, showing a positive impact on the gut microbiota and leading to improvements in adiposity [575]. In a study conducted in postmenopausal women using multi-strain supplementation for a period of 12 weeks, there was no difference in BMI [576].

Knowing the variability of the gut microbiota in humans and the unique interaction that exists between each individual and the microorganisms that inhabit it, the effects of probiotics are still inconsistent. From an extensive analysis of studies conducted in animals, it is clearly perceived that there is great difficulty in replicating the results and this can be explained by several factors such as: differences in study designs, differences in phenotypes of bacteria that can modulate the beneficial activity, even when they belong to the same genus and species, lack of standardization of shelf life (number of live bacteria at expiration), and lack of specification of multi-strain formulations and even of single-strain products [577–579]. Another relevant aspect is the fact that there are different types of formulations/presentations (yoghurts, capsules, sachets, etc.), since the form of presentation of the probiotic can influence its effect.

With regard to human studies, it is also worth to emphasize that comorbidities and cofactors such as age, sex, autoimmune diseases, among others, that are related to changes in the gut microbiota, are not always considered as exclusion criteria, which can influence the results of these investigations [577, 578].

Few studies in humans were capable of replicating the effect observed in animal models, probably due to differences in the composition of the gut microbiota, the interaction between different species of bacteria and their interaction with host cells [558, 580, 581]. There is also a need for well-conducted clinical trials to assess efficacy and safety, preferably placebo-controlled, randomized, double-blind, with adequate sample size and assessment of effect size and clinical relevance [558]. It is important to differentiate association studies from causal relationships. Evidence of causality must be properly investigated.

It should be noted that the microbial diversity of species and strains among individuals is quite variable: each individual has its own pattern of bacterial composition, determined in part by the genotype of the host, and also as a result of initial colonization and by geographic, ethnic and environmental factors, including dietary factors, among others [582].

The actual composition of the gut microbiota is still unknown; there are many bacteria that have not been identified and their action is not understood [558, 583].

Due to variability among probiotics formulations and composition of gut microbiota in humans, as well as the interaction between these factors, studies examining the effect of probiotics supplementation on body weight present conflicting results. Further research is necessary to assess efficacy and safety of different formulations of probiotics.

**Table:** 

Statement	Class of recommendation	Level of evidence
1. There is not enough scientific evidence for the use of probiotic supplementation for weight management	III	A
2. There is not enough scientific evidence for performing gut microbiota tests in clinical practice for the treatment of obesity	III	A

## Behavioral and cognitive therapies and motivational interviewing

Behavioral and cognitive therapies concern therapies that incorporate behavioral and cognitive interventions [584]. This umbrella of approaches can comprise the so called “third wave” therapies, including Acceptance and Commitment Therapy (ACT), Dialectical Behavior Therapy (DBT), Mindfulness-Based Interventions (MBIs), Schema Therapy, and Compassion-Focused Therapy (CFT). These interventions share some of the same components as the “second wave” Cognitive-Behavioral Therapy (CBT) [585].

In addition to the physiology of human energy regulation and pathophysiology of obesity resulting from multiple interacting sources, behavior influences the current weight of the individual, and can also influence the process of weight loss and maintenance [25]. Information on healthy eating is not enough to change dietary behavior, while behavioral treatment provides support for the development of a set of abilities needed to reach a healthy weight [26]. It is known that diet adherence is one of the main barriers of current nutrition interventions for weight management in individuals with overweight and obesity [586].

### Cognitive-behavioral therapy—CBT

CBT is well established for the management of eating disorders (ED) [587]. In a RCT by Dalle Grave et al. [588], CBT was personalized for weight loss and tested in individuals with obesity. The adherence rate at the end of the 18-month study was 76.2%. Patients achieved a mean weight loss of 11.5% after 6 months and 9.9% at the end of the study [588]. A meta-analysis by Jabob et al. [589] showed similar effects. The analysis of 12 studies demonstrated that CBT interventions adapted for weight loss with mean study duration of 10 months led to an average weight loss of − 1.70 kg. This review also identified positive effects on reducing binge eating and emotional eating [589].

Health professionals must be trained to refer patients and psychologists to offer CBT with frequent and individualized contact in the long-term for patients presenting with overweight associated with risk factors or obesity.

### Motivational interviewing—MI

MI is a patient-centered approach with the objective of eliciting commitment to the process of change and is based on the identification and mobilization of intrinsic values (intrinsic motivations) of the individual for the promotion of behavior change. MI is designed to elicit, clarify and solve ambivalence, which strongly influences the process of change, and to positively reinforce the ability of the individual to change behaviors, taking his/her readiness to change into consideration [590–594].

In a systematic review and meta-analysis of 11 RCTs by Armstrong et al. [591], the authors reported greater weight loss in the studies that used MI as a component combined with the intervention [591]. It is worth mentioning that the mean difference in BMI observed in this review was − 0.25 kg/m^2^ e − 1.47 kg in weight [591].

Intervention periods for weight loss longer than 24 months including MI were assessed in a RCT by Vos et al. [595], that studied women with overweight and obesity in primary care followed by 2.5 years, and reassessed between 6 and 7 years after randomization [595]. The authors observed greater weight loss in the intervention group compared with the control group at 6 months, with statistically significant difference; however, this difference became nonsignificant at the end of the study [595].

MI promotes benefits from its association to therapies and interventions for weight loss. Yet, factors such as number of meetings between professionals and patients, duration, and professional training and experience, can influence the effectiveness, possibly explaining the heterogeneity of the findings in the scientific literature [596].

MI can be considered and used by any qualified health professional, combined with interventions and therapies for weight management in patients with overweight and obesity regardless of age, in primary or secondary care.

**Table:** 

Statement	Class of recommendation	Level of evidence
1. Cognitive-Behavioral Therapy should be used for weight management in patients with overweight and obesity	I	A
2. Motivational Interviewing can be considered and used for weight management in patients with overweight and obesity	IIa	A

## Mindful eating 

Mindfulness-Based Eating Awareness Training (MB-EAT), used for the treatment of Binge Eating Disorder [597, 598], has the objectives of developing awareness of the triggers that promote food intake and the physical signals of hunger and satiety, as well as to stop binge eating episodes. The program emphasizes eating pleasure from smaller portions and leads the individual to make healthier food choices [599].

The treatment of obesity is complex and involves reduction in food intake and increase in energy expenditure [600], which can be burdensome, given that individuals with obesity might present greater activation of the hedonic system and suppression of the homeostatic system that controls this process [601]. Mindfulness-Based Interventions (MBIs) can be useful to provide the individuals more resilience, developing greater ability to avoid eating in response to emotions, respecting hunger and satiety cues [600]. Recently, therefore, MBIs have been also studied in the treatment of overweight and obesity [602].

The systematic review conducted by Katterman et al. [603] included the assessment of studies that used MBIs related to disordered eating and weight. The intervention used in the studies ranged from 6 to 16 weeks. The studies that reported significant weight loss were those that focused on weight loss as the primary outcome. Furthermore, one of the studies reported significant weight gain relative to the initial weight after the participation in a Mindfulness-Based Stress Reduction (MBSR) program [603].

Another systematic review and meta-analysis included 18 studies (14 RCTs) that only used official mindfulness-based programs as a primary treatment and weight loss as the primary outcome in adults with overweight and obesity [604]. The authors observed that the studies that combined formal and informal mindfulness practices led to greater weight loss than the studies that used formal practice alone. Besides that, MBIs led to lower average loss of initial weight at the end of the intervention and during follow-up, compared to lifestyle change-based interventions (− 3.3% vs. 4.7% and − 3.5% vs. − 4.3%) [604].

In a systematic review and meta-analysis that evaluated 10 studies that assessed the impact of MBIs on weight loss, it was observed that when the intervention group was compared to a control group, weight loss with MBI was greater than when the control group received no nutritional intervention. On the other hand, when the control group was submitted to nutritional intervention, weight loss was similar in both groups [605].

One of the criticisms mentioned in most of the systematic reviews is that not all of the studies use some validated method to assess mindfulness, making it impossible to know if changes in mindfulness had an actual impact on weight loss [600].

In addition, the diversity in results might be related to the fact that some interventions combined mindfulness programs with other components including nutrition education, physical activity, and behavioral self-monitoring [600]. MBIs used were varied, and the standard protocols of the MBSR or the MB-EAT programs were not always used, but a modified version or a new program that varied concerning the duration of the treatment and practice time [606]. Moreover, it is necessary to investigate if MBIs would bring benefits for weight loss and maintenance in the long term [605].

Despite the promising results, long-term studies are still needed to assess the impact of MBIs on weight loss in men and women with excess weight, including larger samples to ensure that this type of strategy is really effective for the treatment of overweight and obesity.

**Table:** 

Statement	Class of recommendation	Level of evidence
The results related to Mindfulness-Based Interventions in the treatment of obesity are still divergent, and it is yet not possible to guarantee that this is an appropriate approach for all patients	IIa	A

## Weight regain

Body weight and the volume of the adipose tissue result from the balance between energy expenditure and food intake, which, in turn, are influenced both by the individual’s genetic background and the environment. Throughout the evolution process, the ability to store fat was crucial for the survival of our species during periods of severe food scarcity. Nowadays, however, with the abundance of high energy density foods and the intake of an inadequate diet, combined with sedentary behavior, that which once represented an advantage now favors weight gain and an increased prevalence of obesity [607].

The main challenge in the treatment of obesity is to avoid weight regain. The impossibility to keep the weight causes much frustration and, sometimes, leads to discontinuation of the treatment [608].

It is well documented in the literature that regardless of the diet type, weight loss occurs as a consequence of caloric restriction [13–16, 18]. However, most individuals return to their initial weight between 12 and 24 months [13–16, 18]. In the long term, weight maintenance must be supported by the adoption of new food habits and the practice of physical activity, in order to demand less attentional effort from the patient [609]. In fact, it seems to be a characteristic of the individuals who are successful in maintaining weight loss, for whom the duration of weight maintenance is inversely associated with the effort required to maintain it [610].

This is because weight regain is favored by mechanisms that involve multiple actions, both central and peripheric, that are linked with the control of energy expenditure and food intake and aim to reduce energy expenditure and increase energy intake to restore the initial weight. Energy intake and expenditure are regulated by interconnected control mechanisms that adjust themselves to keep the energy stores constant [607].

The hypothalamus integrates signals from different tissues in order to regulate food intake. The secretion of leptin by the adipose tissue reflects the amount of energy stored. On the other hand, orexigenic gastrointestinal-derived hormones such as ghrelin, and anorexigenic hormones such as CCK, GLP-1 and gastric inhibitory peptide (GIP) send information related to the meal, signaling the pre and postprandial state and controlling food intake [611]. Therefore, different afferent signals communicate centrally the long-term status of energy stores (adipose tissue), as well as the energy obtained from the meal, and coordinate the actions needed to keep the energy store [611]. This net of information extends to the mesolimbic system, involved in hedonic eating, which defines eating behavior, the motivation to obtain food and coordinates the reward system [612].

Human studies have shown that, in response to the weight loss induced by caloric restriction, there is a persistent (> 1 year) decrease in the RMR and in TEE, favoring weight regain [613, 614]. This reduction in the resting metabolic rate during weight loss occurs even when lean mass is preserved [615]. Also, studies have shown that individuals who lost weight presented an approximate increase of 20% in muscle energetic efficiency during low-intensity physical activity (assessed by ergospirometry test and by nuclear magnetic resonance spectroscopy of the gastrocnemius muscle). The higher muscle work efficiency using less energy substrate contributes to a significant reduction in TEE [616].

Individuals with obesity submitted to calorie restriction for weight loss presented increased hunger and ghrelin secretion [617]. On the other hand, the levels of CCK [617, 618], GLP-1, GIP and PYY were reduced, even after one year since the beginning of the treatment [617]

These adaptations resulting from weight loss favor weight regain. It was demonstrated that the increased appetite leads to an increase of about 100 kcal/day per kg lost [619].

Studies using functional magnetic resonance in individuals with obesity submitted to weight loss therapy have shown that those who presented higher activation of central areas related to the reward system at the beginning of the treatment lost less weight. In a similar manner, those who presented higher activation were less likely to maintain the weight after 9 months [620].

All of these complex adaptative alterations favor weight regain in order to restore the long-term energy stores. Therefore, the strategies for obesity management should take all the adaptations involved in energy balance into account to help the patient to address the difficulties related to the treatment. Lastly, it is worth to remember both professionals and patients that there are no simple solutions for complex situations, thus wonder diets and products only misinform those involved in the treatment and impair its outcome.

## Conclusions

As presented in this report, several approaches have been developed so far aiming to promote weight loss, however only part of them are based on scientific evidence. Adherence to healthy eating habits comprised in lifestyle changes are one of the cornerstones of obesity treatment. Weight loss is promoted by negative energy balance, therefore low-calorie diet is recommended. Considering that obesity is a chronic disease, individualized diet should be proposed to promote long-term diet adherence and sustainable eating habits. Nutrition practice based on scientific evidence is needed for promoting individual’s global health.

## Data Availability

Not applicable.

## Abbreviations

ABESO: Brazilian Association for the Study of Obesity and Metabolic Syndrome
ACT: Acceptance and commitment therapy
ADF: Alternate day fasting
ADRB2: B2-adrenoreceptor
AHEI: Healthy eating index
ANKRD26: Ankyrin-26
BCAAs: Branched-chain amino acids
BMI: Body mass index
CBT: Cognitive-behavioral therapy
CCK: Cholecystokinin
CFT: Compassion-focused therapy
ChREBP: Carbohydrate responsive element binding protein
CRP: C-reactive protein
CVDs: Cardiovascular diseases
DASH: Dietary approaches to stop hypertension
DBT: Dialectical behavior therapy
DIETFITS: Diet intervention examining the factors interacting with treatment success
DPP: Diabetes prevention program
DPPOS: Diabetes prevention program outcomes study
DSE: Diabetes support and education
ED: Eating disorders
ERICA: Study of cardiovascular risks in adolescents
ESPEN: European Society for Clinical Nutrition and Metabolism
eTRF: Early time restricted feeding
EVCO: Extra-virgin coconut oil
FABP2: Fatty acid binding protein-2
FTO: Fat mass obesity
GBD: Global burden of disease study
GI: Glycemic index
GIP: Gastric inhibitory peptide
GL: Glycemic load
GLP-1: Glucagon-like peptide 1
GRADE: Grading of recommendation assessment, development and evaluation
GWAS: Genome-wide association studies
HCA: Hydroxycitric acid
HSP72: Heat shock protein 72
HTR2C: Serotonin receptor
IF: Intermittent fasting
IKK: Kappa kinase inhibitor
IL-6: Interleukin 6
ILI: Intensive lifestyle intervention
iNOS: Inducible nitric oxide synthase
ISCOLE: International study of childhood obesity, lifestyle and the environment
JECFA: Joint FAO/WHO Expert Committee on Food Additives
kJ: Kilojoules
LCD: Low-calorie diet
LED: Low energy density
LEP: Leptin
LEPR: Leptin receptor
Low-carb: Low-carbohydrate
LPS: Lipopolysaccharides
MB-EAT: Mindfulness-based eating awareness training
MBIs: Mindfulness-based interventions
MBSR: Mindfulness-based stress reduction
MCP-1: Monocyte chemoattraction proteins
MCT: Medium-chain triglycerides
MI: Motivational interviewing
MR: Meal replacements
MyD88: Amyloid differentiation factor 88
NAFLD: Non-alcoholic fatty liver disease
NF-kB: Nuclear factor kappa B
NHANES: National health and nutrition examination survey
NHS: Nurses’ health study
NutriCoDE: Nutrition and chronic diseases expert group
OO: Olive oil
PeNSE: National adolescent school-based health survey
POMC: Proopiomelanocortin
PPAR-γ: Peroxisome proliferator-activated receptor-gamma
PREDIMED: *Prevención con Dieta Mediterránea*
RCT: Randomized clinical trial
REGARDS: REasons for geographic and racial differences in stroke
RMR: Resting metabolic rate
SCFAs: Short-chain fatty acids
SNP: Single nucleotide polymorphism
T1DM: Type 1 diabetes mellitus
T2DM: Type 2 diabetes mellitus
TAB1/2: TGFβ-activated kinase-binding protein
TAK1: TGFβ-activated kinase
TEE: Total energy expenditure
TEI: Total energy intake
TLRs: Toll-like receptors
TNF-α: Tumor necrosis factor alpha
TRAF6: TNF receptor-associated factor 6
UCP1/2: Mitochondrial uncoupling protein-1 and 2
VIGITEL: Surveillance system for risk and protective factors for chronic diseases by telephone survey
VLCD: Very low-calorie diet
VLCKD: Very-low-calorie ketogenic diet
